# Dynamic Fibrous Hydrogels for Stem Cell Homing and In Situ Bone Regeneration

**DOI:** 10.1002/advs.202508803

**Published:** 2025-11-16

**Authors:** Jianmei Chen, Meiling Su, Xinyu Wu, Hongyu Wu, Haotian Wu, Pinghu Zhang, Xueying An, Zongguang Liu

**Affiliations:** ^1^ Key Laboratory of the Jiangsu Higher Education Institutions for Integrated Traditional Chinese and Western Medicine in Senile Diseases Control School of Traditional Chinese Medicine Faculty of Medicine Yangzhou University Yangzhou 225009 P. R. China; ^2^ Microelectronics Industry Research Institute College of Physics Science and Technology Yangzhou University Yangzhou 225009 P. R. China; ^3^ Department of Orthopedic Surgery State Key Laboratory of Pharmaceutical Biotechnology Division of Sports Medicine and Adult Reconstructive Surgery Nanjing Drum Tower Hospital The Affiliated Hospital of Nanjing University Medical School Nanjing 210008 P. R. China

**Keywords:** biomimic, fibrous hydrogel, photothermal stimulation, stem cell homing, tissue regeneration

## Abstract

Stem cell therapy holds great promise for enhancing bone regeneration, but its clinical outcomes are often hampered by ineffective cell homing and compromised paracrine activity in pathological oxidative microenvironments. Here, a dynamic fibrous hydrogel (DFH) is presented, fabricated by covalently crosslinking gelatin with tea‐derived trichomes (TH), which recapitulates the static and dynamic features of the native extracellular matrix (ECM) to enhance stem cell‐mediated bone repair. DFH features a robust, heterogeneous fibrous network that closely mimics the structural complexity and biochemical composition of the ECM, thereby creating a tailored niche for mesenchymal stem cell (MSC) delivery. Specifically, DFH exhibits potent photothermal responsiveness, enabling spatiotemporal regulation that boosts the paracrine secretion of SDF‐1 from encapsulated MSCs, thereby promoting stem cell migration. Furthermore, the polyphenol‐rich TH endows DFH with superior antioxidant capabilities, markedly improving MSC survival under oxidative stress. In murine cranial defects, MSC‐laden DFH (DFH@MSC) with photothermal stimulation enhances stem cell recruitment, promotes angiogenesis, and reduces inflammation, ultimately driving robust bone regeneration. By integrating ECM‐mimetic structural and compositional fidelity with spatiotemporal modulation, DFH introduces a novel “stem cell recruits stem cell” strategy that orchestrates the co‐homing of endogenous and exogenous stem cells, establishing a pioneering therapeutic paradigm for in situ tissue regeneration.

## Graphical abstract

A dynamic fibrous hydrogel (DFH) is developed by covalent crosslinking of gelatin with tea‐derived trichomes, which integrates the structural and compositional fidelity of native extracellular matrix with photothermal and oxidative responsiveness, introducing a novel “stem cell recruits stem cell” strategy that orchestrates co‐homing of endogenous and exogenous stem cells, and establishing a pioneering therapeutic paradigm for in situ tissue regeneration.

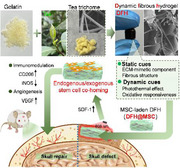

## 1 Introduction

Bone defects, arising from traumatic injuries or pathological conditions such as osteosarcoma, osteoporosis, and femoral head necrosis, represent a significant global health challenge. The global prevalence of bone defects has risen sharply in recent decades, with 450 million bone fracture cases reported in 2019, marking a 70% increase since 1990.^[^ ^1^ ^]^ In China, over 6 million patients were diagnosed with bone defects or dysfunction in 2021, and ≈4 million of these individuals required bone grafts or biomaterial‐based scaffolds to provide mechanical support and osteogenic microenvironments.^[^ ^2^ ^]^ Critically, these defects create a hostile microenvironment characterized by persistent oxidative stress, chronic inflammation, and compromised vascularization, which severely hinders endogenous healing and limits the efficacy of conventional therapies.^[^ ^3^, ^4^ ^]^

Mesenchymal stem cells (MSCs) represent a promising therapeutic strategy for bone regeneration due to their paracrine activity,^[^ ^5^ ^]^ which involves the secretion of a range of growth factors, cytokines, and extracellular vesicles that not only stimulate new blood vessel formation to ensure an adequate blood supply but also modulate the immune response and reduce inflammation, creating a favorable microenvironment for injured tissue healing.^[^ ^6^ ^]^ However, the clinical translation of MSC therapy faces critical challenges in bone defect repair. Transplanted MSCs exhibit poor homing efficiency, and their survival and paracrine function are further compromised by the oxidative and inflammatory microenvironment, drastically reducing therapeutic efficacy. Therefore, there is an urgent need for advanced delivery platforms that can protect MSCs, enhance their retention, and sustain their regenerative functions in pathological conditions.

In native tissues, cells engage with the dynamic extracellular matrix (ECM), which delivers both static structural support and spatiotemporal biochemical regulation to direct cellular behavior,^[^ ^7^ ^]^ thereby inspiring the development of ECM‐mimetic biomaterials for enhanced stem cell therapy and tissue regeneration.^[^ ^8^, ^9^, ^10^, ^11^, ^12^ ^]^ Among these, fibrous hydrogels, engineered as 3D networks of prefabricated fibers or polymeric materials, emerge as a breakthrough platform by recapitulating the native ECM's anisotropic organization and biomechanical properties, thereby offering unprecedented opportunities for cell delivery and tissue reconstruction.^[^ ^13^, ^14^, ^15^, ^16^ ^]^ For instance, peptide fiber‐reinforced hydrogels potentiate mechanotransduction and cellular metabolism, promoting osteogenic differentiation and bone regeneration,^[^ ^26^ ^]^ while silk fibroin methacrylate hydrogels incorporated into PLGA scaffolds establish a hydrated 3D niche that markedly enhances MSC viability and lineage commitment.^[^ ^27^, ^28^ ^]^ Despite notable advancements, prevailing fibrous hydrogels remain fundamentally limited by their inability to dynamically adapt to pathological cues, including oxidative stress and inflammatory signals, resulting in compromised cellular performance and unsatisfactory regenerative outcomes.^[^ ^17^, ^18^, ^19^ ^]^

**Scheme 1:**
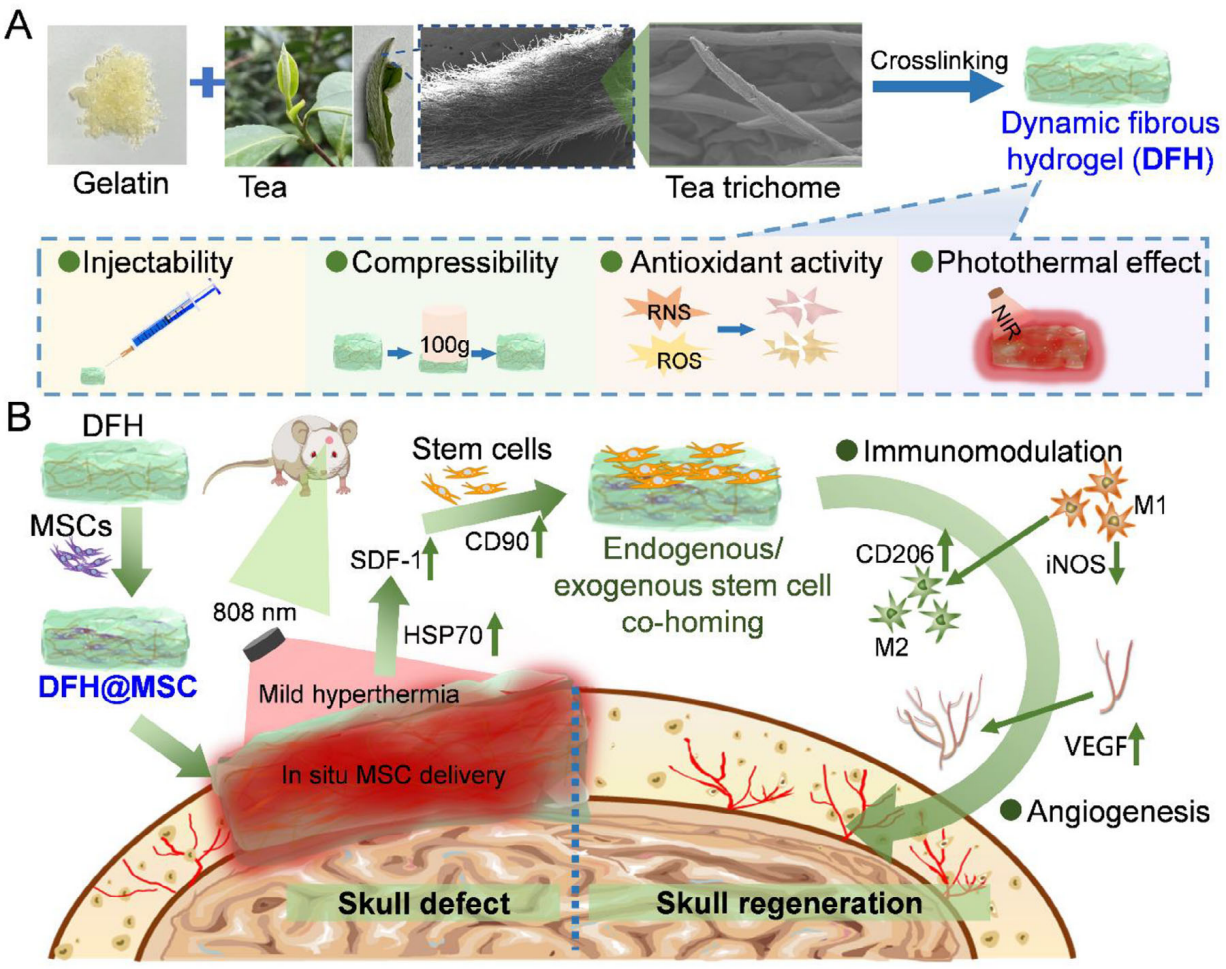
Engineered dynamic fibrous hydrogel (DFH) for augmented bone regeneration. A) DFH fabrication via covalent crosslinking of gelatin and tea trichomes (TH) yields an injectable matrix with high compressibility, robust antioxidant activity, and tunable photothermal responsiveness. B) Therapeutic mechanism of MSC‐laden DFH (DFH@MSC). DFH@MSC synergistically combines ECM‐mimetic cues with photothermal‐ and redox‐responsiveness to simultaneously enhance stem cell retention, bioactivity, and endogenous recruitment, thereby amplifying immunoregulation, potentiating angiogenesis, and driving efficient bone defect regeneration.

Here, we engineer an injectable dynamic fibrous hydrogel (DFH) as a multifunctional platform designed to enhance MSC therapy by optimizing cell retention, functional activation, and microenvironmental modulation for effective bone regeneration. DFH is formed through covalent crosslinking between gelatin (an ECM‐mimetic component) and tea trichomes (TH, isolated from the tea plant), faithfully replicating both the static and dynamic characteristics of the native ECM. The biomimetically designed DFH integrates three therapeutic functions. First, DFH possesses superior antioxidant capability, as the polyphenol‐rich TH component effectively scavenges reactive oxygen species (ROS), thereby protecting encapsulated MSCs from oxidative damage. Second, it holds immunomodulatory potential, as the ECM‐mimetic niche works synergistically with MSC paracrine factors to attenuate inflammation and promote macrophage polarization toward the pro‐regenerative M2 phenotype. Third, DFH enables photothermal‐activated stem cell recruitment. Its strong photothermal responsiveness allows spatiotemporal control over the release of chemokines, such as SDF‐1, from MSCs, enhancing the homing of endogenous stem cells. Additionally, DFH demonstrates rapid gelation, high mechanical strength, and excellent biocompatibility, supporting its potential for clinical translation (**Scheme** **1**A). In a murine cranial defect model, MSC‐encapsulated DFH (DFH@MSC) combined with photothermal stimulation potentiated SDF‐1 release, enhancing stem cell homing and recruitment while amplifying paracrine‐mediated immunomodulation and angiogenesis, collectively driving robust in situ bone regeneration (Scheme 1B). Overall, by coupling ECM‐mimetic structural and compositional fidelity with photothermal and oxidative responsiveness, DFH provides a comprehensive strategy to protect MSCs, resolve inflammation, and orchestrate stem cell co‐homing for efficacious cranial repair.

## 2 Results

### 2.1 Preparation and Characterization of DFH

**Figure 1:**
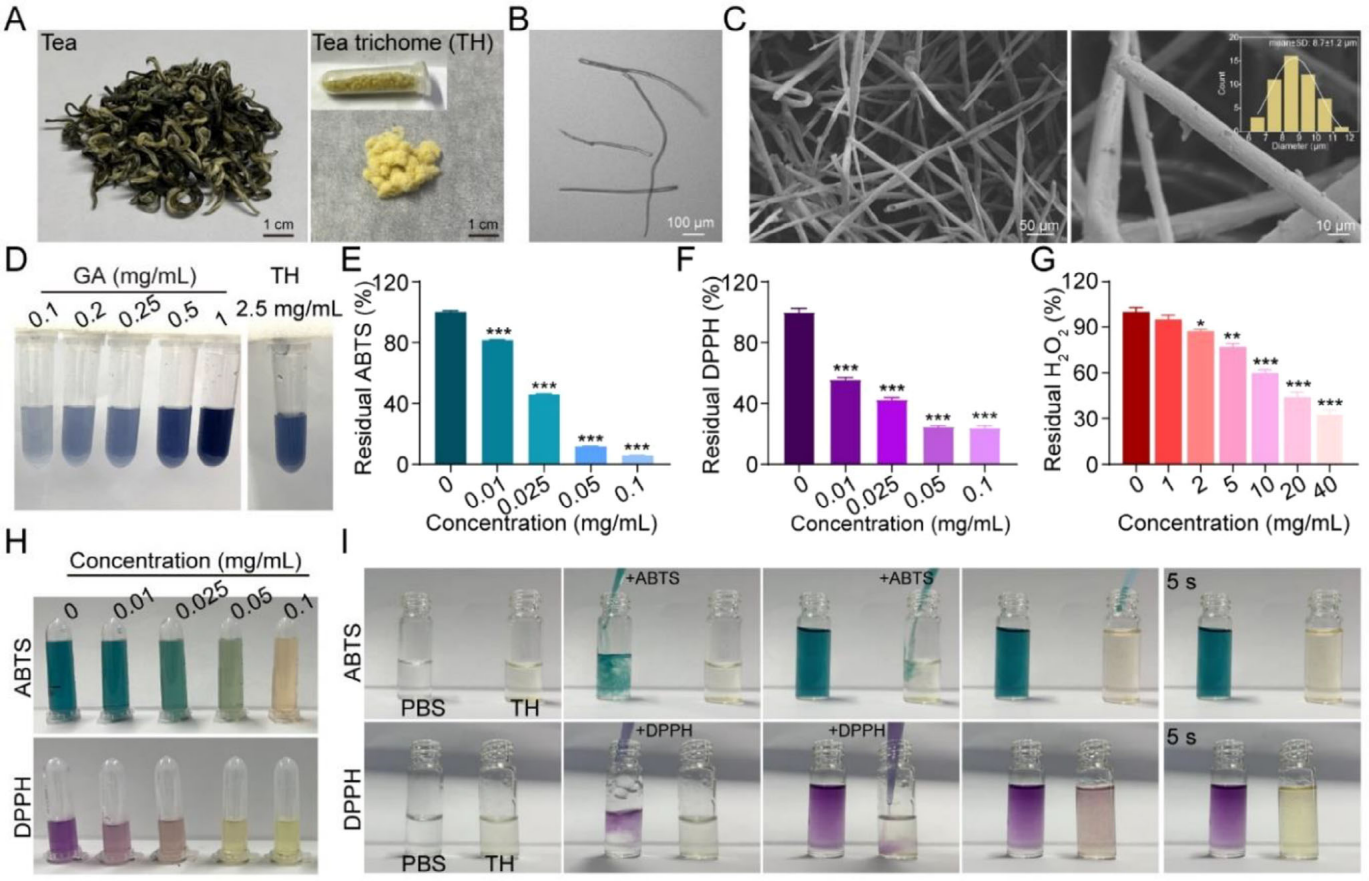
Structural and functional characterization of TH. A) Digital photographs of tea leaves and TH. B) Optical image of TH observed under an inverted light microscope. C) SEM images of TH. Inset: diameter distribution of TH. D) Quantification of total polyphenol content in TH by Folin‐Ciocalteu assay. GA: gallic acid (standard reference). E)‐G) Free radical scavenging capability of TH against ABTS^+^·, DPPH· and H_2_O_2_. Data are expressed as mean ± SD (n = 5). ^*^ *p*<0.05, ^**^ *p*<0.01, ^***^ *p*<0.001 versus control group (0 mg mL^−1^). H) Color changes of ABTS and DPPH solutions after incubation with varying TH concentrations. I) Real‐time decolorization kinetics of ABTS and DPPH solutions after incubation with TH.

Natural materials have become indispensable for cell delivery and tissue engineering, owing to their intrinsic biocompatibility and bioactivity. TH, also referred to as “Cha Hao” in Chinese, is enriched in the buds and young leaves of tea plants (**Figure** **1**A), contributes to the unique flavor and high quality of tea products, and provides physical and biochemical defenses for tea plants.^[^ ^20^ ^]^ Structural analyses via inverted light microscopy and scanning electron microscopy (SEM) revealed that TH possesses an unbranched fibrous structure with uniform diameters averaging 8.7 µm (Figure 1B,C), suggesting its suitability for engineering fibrous hydrogels that mimic native ECM's fibrous microstructure and mechanical properties. Quantitative analysis using the Folin‐Ciocalteu assay demonstrated a high polyphenol content in TH (≈180 µg mg^−1^) (Figure 1D; Figure S1, Supporting Information), underscoring its potential as a multifunctional, antioxidant‐rich biomaterial. The antioxidant capacity of TH was systematically evaluated using three free radical scavenging assays (ABTS^+^·, DPPH·, and H_2_O_2_).^[^ ^21^ ^]^ TH demonstrated concentration‐dependent scavenging efficiencies against 2,2′‐azino‐bis (3‐ethylbenzothiazoline‐6‐sulfonic acid) (ABTS), 2,2‐diphenyl‐1‐picrylhydrazyl (DPPH) radicals, and hydrogen peroxide (H_2_O_2_) (Figure 1E–G; Figure S2, Supporting Information). It is noteworthy that TH demonstrated immediate scavenging activity against ABTS^+^· and DPPH·, visually confirmed by rapid decolorization of the ABTS (blue‐green) and DPPH (purple) solutions after mixing with TH (Figure 1H,I; Videos S1 and S2, Supporting Information). Together, these results position TH as a structurally and functionally dual‐advantage biomaterial, merging polyphenolic bioactivity with ECM‐mimetic topography for regenerative applications.

**Figure 2:**
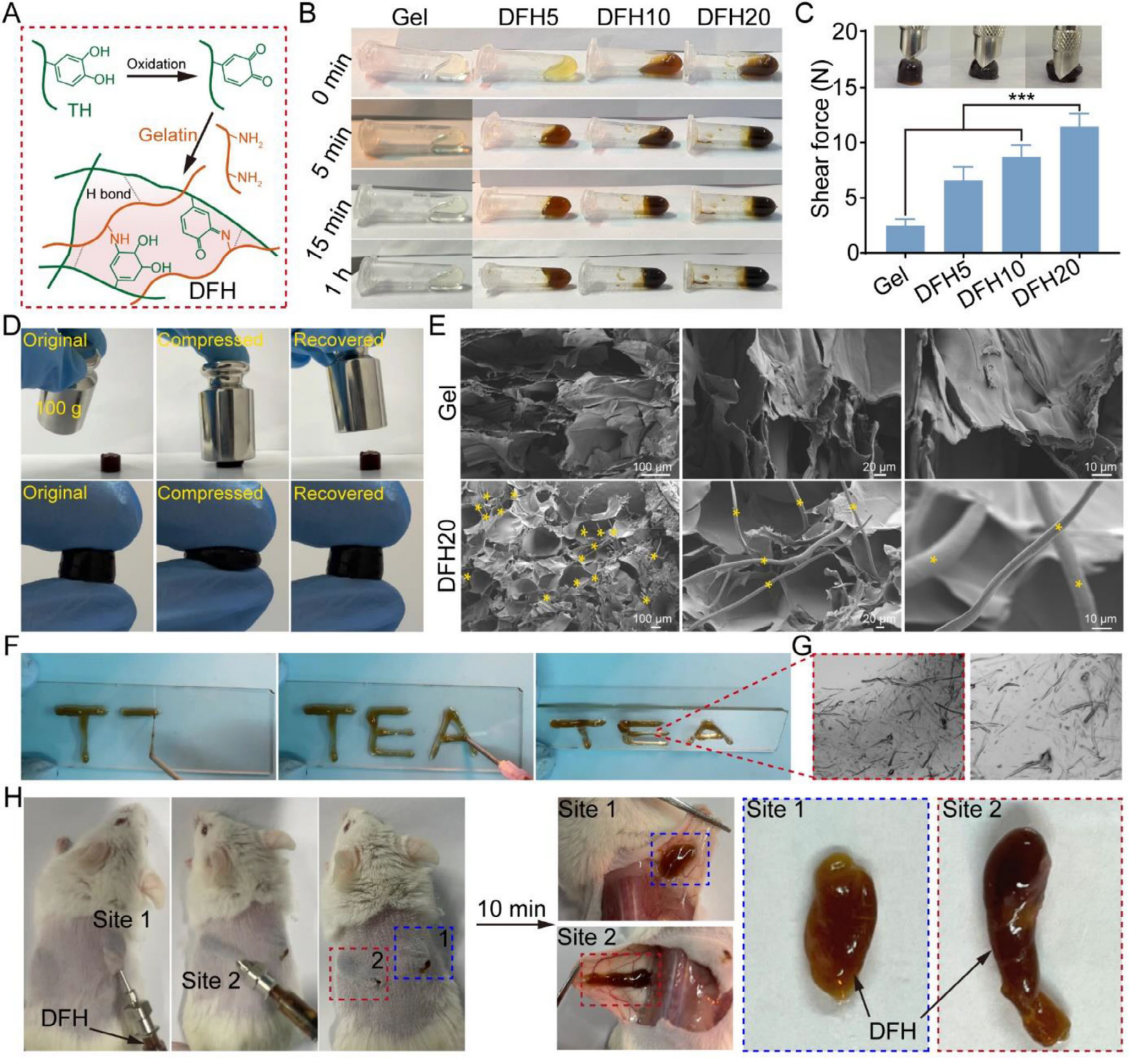
Preparation and characterization of DFH. A) Schematic of covalent crosslinking between amino groups of gelatin and oxidized quinone moieties derived from TH. B) Representative images of DFH fabrication process. C) Shear strength of DFH with varying TH concentrations (n = 5). D) Compressive performance of DFH. E) SEM images of DFH. Asterisks (*) indicate TH encapsulated in the hydrogel. F) Injectability of DFH through an 18G syringe. G) Optical microscopy images of TH distribution in DFH matrix. H) In vivo structural maintenance of DFH after subcutaneous implantation.

To faithfully recapitulate the fibrous microstructure and biochemical composition of ECM, a dynamic fibrous hydrogel (DFH) was engineered via covalent crosslinking of TH with gelatin, which integrates the fibrous structure of TH with the bioactive properties of gelatin, a collagen derivative widely used to mimic ECM components due to its cell‐adhesive RGD motifs. Mechanistically, under alkaline or neutral conditions, TH is converted to oxidized TH (oTH), generating reactive quinone groups, which subsequently react with nucleophilic amino acid residues (e.g., lysine and arginine) in gelatin via Schiff base formation or Michael addition, resulting in stable covalent linkages (**Figure** **2**A).^[^ ^22^, ^23^ ^]^ The chemical transformation of TH to oTH was confirmed by UV‐Vis‐NIR spectra, which revealed a distinct increase in absorbance at ≈350 nm (Figure S3A, Supporting Information), consistent with the oxidation of polyphenols to quinones.^[^ ^24^ ^]^ Correspondingly, oTH displayed enhanced absorbance in the region, accompanied by a darkening of the solution color (Figure S3B, Supporting Information). The mechanism behind these phenomena can be attributed to the large number of conjugated functional groups within the structure of TH quinone molecules, allowing 𝜋‐𝜋 molecular interactions and strong conjugation to form among TH quinone molecules, which endows TH quinones with unique potential for photothermal conversion.^[^ ^25^ ^]^ Furthermore, in comparison to the FTIR spectra of TH, the peak intensity of oTH at 3200–3700 cm^−1^ shifted to a broader band after the oxidation reaction (Figure S3C, Supporting Information).^[^ ^26^ ^]^ The most likely explanation is that the hydroxyl groups were involved in the oxidation polymerization of catechol, which caused the change in the hydroxyl vibration bands. Additionally, the characteristic peak of the quinone structure appeared at 1655 cm^−1^, indicating that the phenolic hydroxyl groups of TH had been oxidized to active quinones.^[^ ^27^, ^28^ ^]^

The crosslinking kinetics were precisely controlled using sodium periodate (SP), a potent oxidizer that accelerates polyphenol oxidation.^[^ ^23^, ^29^ ^]^ The critical role of SP in this process was confirmed by control experiments, where gelatin‐TH mixtures without SP failed to form stable hydrogels (Figure 2B; Figure S4, Supporting Information). Notably, the hydrogel formation kinetics were tightly regulated by the concentration of TH, with the gelation time decreasing precipitously from 1 h for DFH5 (5% TH) to 5 min for DFH20 (20% TH).

FTIR analysis was conducted to understand the hydrogel's formation mechanism. As shown in Figure S5A (Supporting Information), the peak near 3300 cm^−1^ broadened and shifted to 3278 cm^−1^ in DFH relative to gelatin (3280 cm^−1^) due to hydrogen bonding between gelatin and polyphenols of TH.^[^ ^30^, ^31^ ^]^ Notably, the C═N stretching vibrations of Schiff base groups (1600‐1650 cm^−1^) overlapped with the amide I band (1627 cm^−1^) of gelatin, resulting in peak shifts to 1630 cm^−1^ in DFH (Figure S5B, Supporting Information).^[^ ^32^, ^33^, ^34^ ^]^ Concurrently, the amide III band (C‐N stretching coupled with bending) shifted from 1236 cm^−1^ in gelatin to 1238 cm^−1^ in DFH,^[^ ^35^ ^]^ and the peak at 1446 cm^−1^ (aromatic C‐N stretching or CH_2_ bending modes) intensified and shifted to higher frequencies (1448 cm^−1^) in DFH, suggesting Michael addition reaction between TH‐derived quinones and gelatin amino groups.^[^ ^34^ ^]^ Thus, the oxidation of the polyphenolic hydroxyl groups in TH forms quinone groups, which react with nucleophilic amino acid residues (e.g., lysine, arginine) in gelatin via Schiff base or Michael addition reactions,^[^ ^29^, ^33^ ^]^ while the phenolic groups in TH act as hydrogen donors for the carboxyl groups in gelatin.^[^ ^30^, ^31^, ^36^, ^37^ ^]^ Overall, non‐covalent (hydrogen bonds) and covalent interactions (Schiff base and Michael addition) collectively stabilize the hydrogel structure.

The resulting fibrous hydrogel system not only creates an ECM‐mimetic microenvironment conducive for cell survival and tissue regeneration, but also restores mechanical integrity, critical for load‐bearing applications such as skull repair. The mechanical properties of the DFH were tested across varying TH concentrations. Shear strength measurements demonstrated a concentration‐dependent enhancement, escalating from 2.49 N for DFH5 to 11.5 N for DFH20 (Figure 2C). A 100 g weight or fingertip was used to compress the DFH to qualitatively demonstrate its elastic response. Upon removal of the applied pressure, the hydrogel rapidly returned to its original shape, confirming its excellent deformability and resilience (Figure 2D; Videos S3 and S4, Supporting Information). This tunable mechanical performance enables DFH to meet diverse clinical requirements, while its superior compressive resilience offers potential protection against secondary brain injury during skull regeneration. SEM images revealed the porous architecture of DFH, characterized by anisotropic fibrous TH alignment integrated within a 3D network (Figure 2E), biomimetically replicating the structural anisotropy of native ECM.

Injectable hydrogels represent a particularly advantageous approach for treating irregularly shaped bone injuries, as they promote seamless integration with adjacent tissues while minimizing invasiveness during surgery.^[^ ^38^ ^]^ Owing to its rapid gelation kinetics and superior mechanical performance, DFH20 was selected for further investigation. The injectability of DFH was tested by loading the pre‐gel solution into a syringe and extruding it through an 18G nozzle. As shown in Figure 2F and Video S5 (Supporting Information), DFH maintained structural integrity post‐extrusion, and the fibrous morphology and uniformity of TH within the hydrogel were observed in the extruded filaments under an inverted phase‐contrast microscope (Figure 2G). Notably, following subcutaneous injection in mice, rapid in situ gelation occurred within 10 min (Figure 2H), validating its injectability and clinical translation potential.

The engineered DFH strategically integrates TH's fibrous architectures with gelatin's bioactivity, faithfully reconstructing both the structural framework and biochemical signaling of native ECM to create an ideal microenvironment for MSC delivery. As shown in Figure S6 (Supporting Information), encapsulated MSCs (DFH@MSC) initially adopt spheroid morphologies before transitioning to spread phenotypes within 3 days, demonstrating the hydrogel's permissive nature for cell growth. The water absorption capacity of DFH was quantified via gravimetric analysis,^[^ ^39^ ^]^ revealing an equilibrium water absorption of 196±5.6% in PBS (pH 7.4, 37 °C) (Figure S7A, Supporting Information). Furthermore, the swelling ratio reached a maximum of 54.7±5.4% under the same conditions (Figure S7B, Supporting Information), classifying DFH as a non‐swelling hydrogel that retains its original shape in an aqueous environment, a critical feature for reliable cell delivery and tissue regeneration applications.^[^ ^39^ ^]^

### 2.2 Redox‐ and Photothermal Responsiveness of DFH

**Figure 3:**
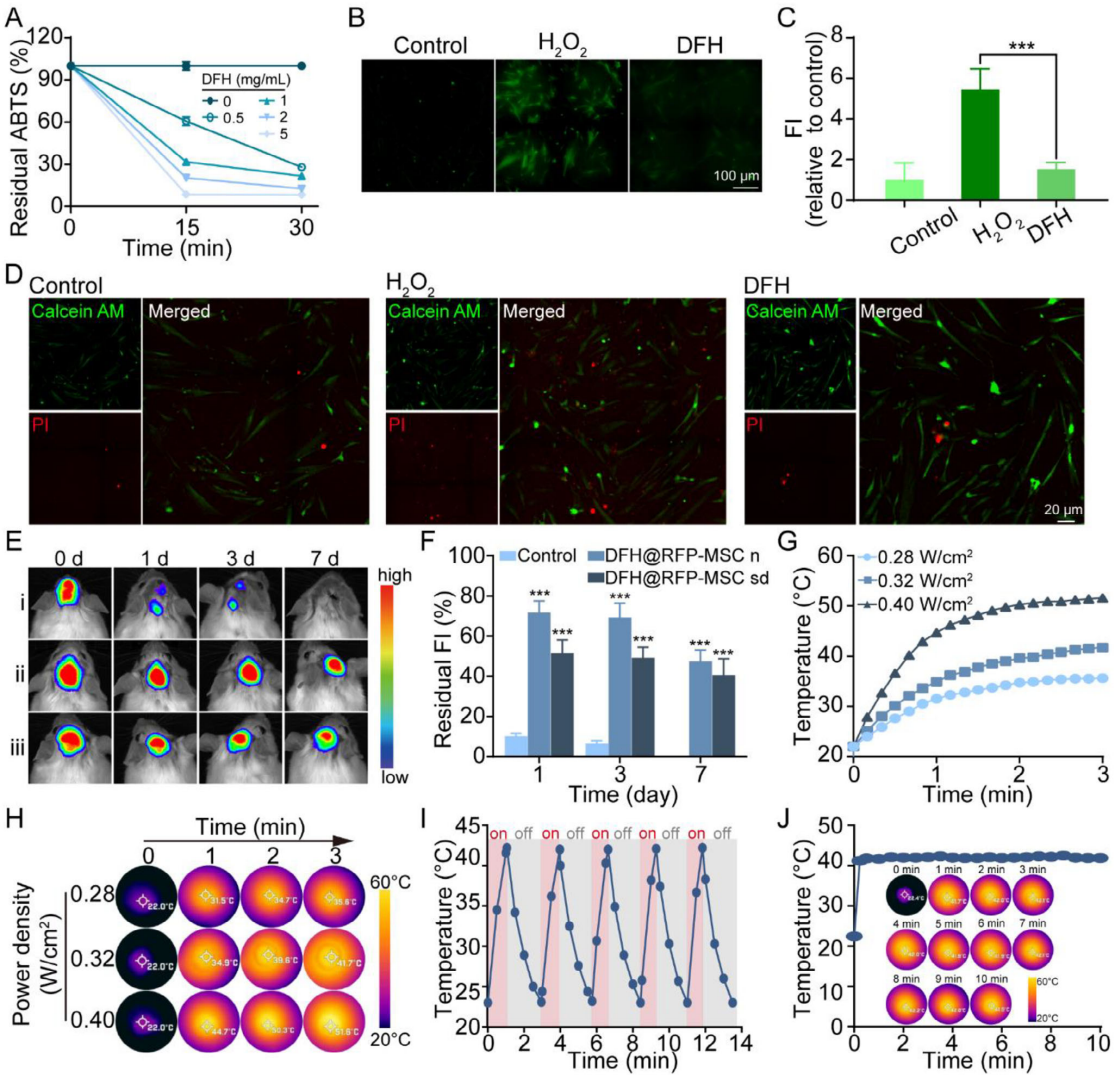
Antioxidant and photothermal characterization of DFH. A) ABTS^+^· scavenging activity of DFH at varying concentrations (n = 5). B)‐C) Fluorescence images and quantitative analysis of intracellular reactive oxygen species (ROS) levels in all experimental groups (n = 5). D) Live/dead staining of MSCs cultured with or without DFH in an oxidative stress microenvironment. E) In vivo viability of RFP‐labeled MSCs (RFP‐MSCs) encapsulated in DFH (DFH@RFP‐MSC) after one‐week subcutaneous implantation in both normal and skull defect murine models. i: RFP‐MSC (control); ii: DFH@RFP‐MSC in normal mice (DFH@RFP‐MSC n); iii: DFH@RFP‐MSC in skull defect mice (DFH@RFP‐MSC sd). F) Quantification of fluorescence intensity (FI) (n = 5). G) Photothermal response kinetics of DFH under 808 nm NIR irradiation at varying power densities. H) Real‐time infrared thermal images of DFH during NIR irradiation. I) Photothermal cyclic performance of DFH under repeated on/off NIR irradiation. J) Photothermal stability of DFH during continuous 10‐min irradiation.

After establishing ECM‐mimetic static cues (structural and compositional fidelity), dynamic responsiveness to microenvironmental stimuli was incorporated into DFH design. Oxidative stress, characterized by excessive ROS generation, is a critical barrier in injured tissues, causing MSC viability loss, impaired biofunctions, and delayed tissue repair. The oxidative responsiveness of DFH was first investigated by testing its radical scavenging efficiency against ABTS⁺·. As shown in **Figure** **3**A, DFH exhibited time‐ and concentration‐dependent scavenging activity against ABTS^+^·, as evidenced by rapid decolorization of the ABTS (blue‐green) solution (Figure S8, Supporting Information). Even after one‐week subcutaneous implantation, DFH retained substantial antioxidant capacity (67.0% relative to pristine levels), with the decrease in ABTS‐scavenging activity likely attributed to the release and consumption of antioxidant components (Figure S9, Supporting Information). To assess intracellular ROS levels, H_2_O_2_‐exposed MSCs were used as an oxidative stress injury model, and the ROS levels were detected using the DCFH‐DA fluorescent probe. As shown in Figure 3B,C, H_2_O_2_ stimulation induced an intense green fluorescence signal, indicative of significant ROS production. In contrast, DFH treatment reduced DCFH‐DA fluorescence intensity by 72%, demonstrating robust ROS scavenging. Calcein AM/PI staining further confirmed that DFH‐treated MSCs maintained high viability without cytotoxicity (Figure 3D).

To evaluate DFH's cytoprotective capability in vivo, MSCs were genetically labeled with red fluorescent protein (RFP) using a lentivirus‐mediated transfection kit, establishing a stable RFP‐expressing cell line (Figure S10, Supporting Information). The RFP‐MSCs were encapsulated within DFH (DFH@RFP‐MSC) and implanted into the murine scalp with or without skull defects. Fluorescence intensity was monitored over a one‐week period using an in vivo imaging system. As shown in Figure 3E,F, significantly higher fluorescence intensity was observed in both skull‐defect and healthy murine models compared to the control group, demonstrating the robust cytoprotective effect of DFH in physiological conditions. These results emphasize the superior antioxidant activity of DFH, which mitigates oxidative stress‐induced cell damage, thereby improving cell delivery and tissue repair in complex defect environments.

External stimuli, such as electric fields, mechanical deformation, light, magnetic fields, and temperature, can dynamically modulate cellular behavior without damaging the cells, triggering changes that promote tissue regeneration.^[^ ^7^ ^]^ Photothermal stimulation offers particular advantages due to its non‐invasive properties, deep tissue penetration, and precise thermal control, demonstrating significant potential for regulating cellular activity, behavior, and functions, particularly in accelerating bone and skin regeneration. Oxidized polyphenols exhibit intrinsic photothermal properties due to the formation of conjugated quinone structures during oxidation, which enhance light absorption and thermal conversion efficiency,^[^ ^25^ ^]^ positioning DFH as a promising photothermal platform for modulating cell fate. To evaluate the photothermal performance of DFH, real‐time thermographic analysis was conducted under 808 nm near‐infrared (NIR) irradiation at incremental power densities, a wavelength selected for its optimal tissue penetration depth.^[^ ^40^ ^]^ As shown in Figure 3G,H, DFH displayed a power‐dependent temperature response, achieving a temperature surge from an ambient 22 °C to 41.7 °C at 0.4 W cm^−2^ within 3 min. Moreover, DFH exhibited excellent photothermal stability, maintaining consistent performance not only over five consecutive on/off cycles (Figure 3I) but also one week after subcutaneous implantation (Figure S11, Supporting Information). It also demonstrated the ability to maintain therapeutic hyperthermia at ≈41 °C for 10 min under continuous irradiation (Figure 3J). This precise thermal controllability minimizes the risk of thermal injury to cells or adjacent tissue through programmed irradiation protocols.^[^ ^41^ ^]^

The effects of photothermal stimulation on MSC biofunctions were investigated through qRT‐PCR, which revealed a significant upregulation of VEGF under DFH‐mediated photothermal activation (Figure S12, Supporting Information). As a key angiogenic mediator secreted by MSCs, VEGF not only attenuates apoptosis both in vitro and in vivo but also promotes cell proliferation and differentiation,^[^ ^42^ ^]^ playing a crucial role in accelerating tissue repair.^[^ ^43^ ^]^ The photothermally enhanced MSC functions were consistent with previous studies,^[^ ^44^ ^]^ confirming the capability of DFH to deliver spatiotemporally controlled photothermal stimulation that improves cell viability and amplifies therapeutic benefits through synergistic paracrine signaling. Taken together, the dual‐biomimetic hydrogel DFH integrates static and dynamic cues of ECM, not only enhancing MSC in vivo retention and preserving their viability under oxidative stress but also amplifying their paracrine effects, indicating improved therapeutic efficacy for in situ bone regeneration.

### 2.3 Degradation and Biocompatibility of DFH

**Figure 4:**
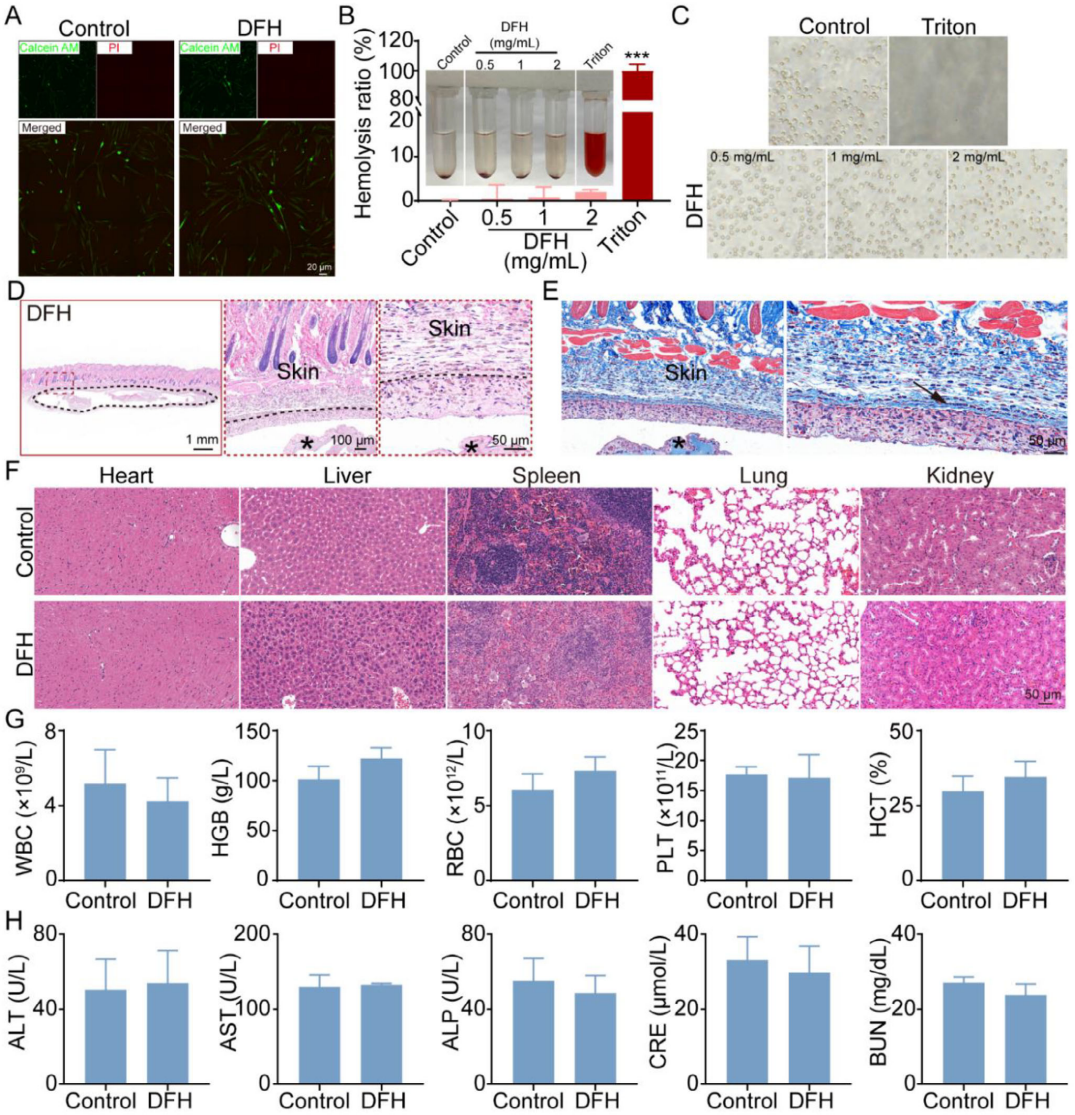
Biocompatibility of DFH. A) Live/dead staining of MSCs cultured with DFH extract for 24 h. B) Quantitative hemolysis analysis. Inset: representative macroscopic images of the blood after 1‐h incubation with PBS (negative control), DFH, and Triton (positive control) (n = 5). C) Erythrocyte morphology after incubation with DFH. D) H&E and E) Masson staining of implanted hydrogel‐adjacent tissues at 4 weeks post‐implantation. Dashed black lines mark the margin of implanted DFH. Asterisks indicate the residual hydrogels. The black arrow indicates the fibrous capsule. F) H&E staining of major organs at 4 weeks post‐implantation. G) Blood routine analysis at 4 weeks post‐implantation (n = 3). H) Serum levels of liver function markers (ALT, AST, ALP) and renal function markers (CRE, BUN) in mice at 4 weeks post‐implantation (n = 5).

To evaluate the therapeutic potential and clinical translatability of DFH, comprehensive assessments of its biocompatibility, including cytocompatibility, hemocompatibility, in vivo safety, and degradation profile, were conducted. Live/dead staining revealed dense green fluorescence (live cells) with minimal red fluorescence (dead cells) in the DFH group (**Figure** **4**A), indicating excellent cytocompatibility. Hemocompatibility tests revealed minimal hemoglobin released in the DFH group, comparable to the PBS negative control, while the Triton positive control showed significant hemolysis (Figure 4B). The hemolysis rate of DFH was below 5%, as confirmed by spectrophotometric analysis and phase‐contrast microscopy, which showed intact erythrocytes in DFH‐treated samples (Figure 4C).

Subcutaneous implantation in mice demonstrated that DFH maintained more than 80% of its initial mass after one week, confirming satisfactory short‐term stability (Figure S13, Supporting Information). Gradual degradation was observed, with minimal residual hydrogel remaining by week 4, corresponding to the slower‐degrading TH component. Macroscopic observation revealed no adverse reactions, such as swelling, erythema, or ulceration, in DFH‐implanted mice (Figure S14, Supporting Information). Histological analysis using H&E staining at week 1 showed transient neutrophil infiltration (Figure S15A–C, Supporting Information), consistent with a normal initial foreign body response. Immunofluorescence staining for F4/80 confirmed macrophage density comparable to the control group (Figure S15D, Supporting Information). By week 4, H&E staining demonstrated normal tissue architecture at the implant interface, with no significant cell aggregation (Figure 4D). The fibrous capsule thickness surrounding the implant decreased progressively from 140.6 µm (week 1) to 37.1 µm (week 4) (Figure 4E; Figure S15E, Supporting Information), indicating successful resolution of acute inflammation and transition to the tissue remodeling phase.

Furthermore, histopathological examination of major organs (heart, liver, spleen, lung, and kidney) revealed no pathological alterations or evidence of systemic toxicity (Figure 4F). Hematological analysis provided additional evidence supporting the biocompatibility of DFH, with all key parameters, including white blood cell (WBC) count, red blood cell (RBC) count, hemoglobin concentration (HGB), hematocrit (HCT), and platelet (PLT) count, remaining within normal ranges and comparable to the control group (Figure 4G; Figure S15F, Supporting Information). Serum biochemical analysis evaluated hepatic and renal functions following the 4‐week implantation. No significant differences were observed in the serum levels of alanine aminotransferase (ALT), aspartate aminotransferase (AST), alkaline phosphatase (ALP), creatinine (CRE), or blood urea nitrogen (BUN) between the control and DFH groups (Figure 4H). Collectively, these results indicate that DFH exhibits excellent biocompatibility, controlled degradation, and minimal immunogenicity, supporting its potential for clinical translation.

### 2.4 DFH@MSC Enhanced Bone Regeneration

**Figure 5:**
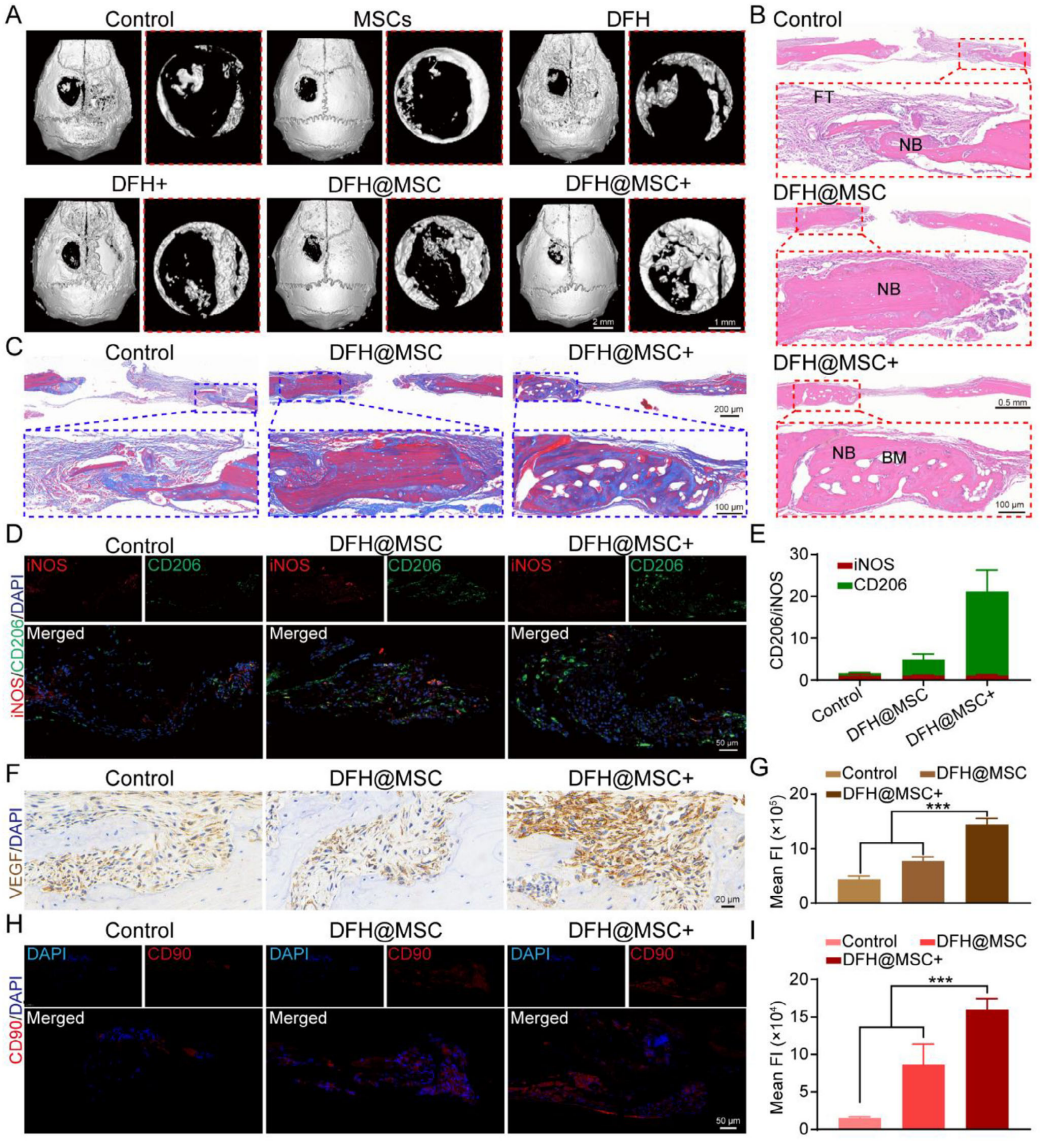
Therapeutic benefits of DFH@MSC with photothermal stimulation (DFH@MSC+) in skull regeneration. A) 3D reconstructed micro‐CT images of murine skull defects at 2 weeks post‐surgery. B) H&E staining of the skull defects. FT: fibrous tissue; NB: newly formed bone tissue; BM: newly formed bone marrow cavity. C) Masson's trichrome staining. D)‐E) Immunofluorescence staining images for iNOS and CD206. F)‐G) Immunohistochemical staining for VEGF expression. H)‐I) Immunofluorescence staining for CD90‐positive cells.

A murine calvarial defect model was employed to assess the bone regenerative capacity of DFH@MSC with or without NIR photothermal stimulation, denoted as DFH@MSC+ and DFH@MSC, respectively. For the photothermal stimulation group, NIR irradiation was applied to maintain a localized temperature of ≈42 °C, a threshold demonstrated to regulate cell fate and promote tissue repair without inducing thermal injury.^[^ ^44^ ^]^ Micro‐CT analysis at 2 weeks post‐implantation demonstrated significantly enhanced new bone formation in the DFH@MSC+ group, contrasting with minimal repair in the blank control group (**Figure** **5**A). Quantitative analysis showed that DFH@MSC+ treatment resulted in the highest defect closure rate (77.8%), followed by DFH@MSC (60.1%), DFH+ (53.7%), MSCs (40.0%), DFH alone (37.8%), and untreated controls (32.0%) (Figure S16, Supporting Information). This superior regeneration was further supported by the observation that DFH@MSC+ exhibited the highest values in bone volume/tissue volume (BV/TV) and trabecular number (Tb. N) among all experimental groups (Figure S17, Supporting Information). Building on these findings, histological examination provided deeper insights into the regenerative process, with H&E staining confirming substantial new bone (NB) deposition in the DFH@MSC+ group, in contrast to predominant fibrous connective tissue (FT) in the control (Figure 5B). Notably, DFH@MSC+ treatment resulted in complete defect healing with newly formed bone marrow cavities (BM), while Masson's trichrome staining demonstrated an abundant collagen‐rich bone matrix at the defect site (Figure 5C), indicating active bone remodeling and regeneration.

To evaluate local immune responses, immunofluorescence staining for CD206 (M2 macrophage marker) and iNOS (M1 macrophage marker) was performed at 3 days post‐surgery. Quantitative analysis revealed a 4.4‐ and 21.2‐fold increase in CD206^+^ cells in the DFH@MSC+ group compared to the DFH@MSC and the control groups, respectively, with minimal iNOS+ cells observed (Figure 5D,E; Figure S18, Supporting Information). The results demonstrate that DFH@MSC alleviates inflammation, and DFH@MSC combined with NIR irradiation further enhances M2‐type polarization of macrophages, fostering a regenerative immune microenvironment for bone regeneration.^[^ ^38^ ^]^ Immunohistochemical staining for VEGF confirmed a higher VEGF‐positive level in the DFH@MSC+ group than that in the DFH@MSC and control groups, showing a larger area of brown and/or reddish‐brown staining evenly distributed within the defect areas (Figure 5F,G). These findings suggest that the synergistic effects of mild photothermal therapy and DFH@MSC not only induce macrophage M1 to M2 phenotype conversion but also enhance angiogenesis, which is essential for bone healing.

To investigate MSC homing, CD90^+^ cell localization was analyzed by immunofluorescence staining. At 2 weeks post‐implantation, the DFH@MSC+ group showed 1.8‐ and 8.0‐fold higher CD90^+^ cell density compared to the DFH@MSC and control groups, respectively (Figure 5H,I), demonstrating that DFH@MSC with moderate photothermal stimulation promotes MSC homing. Given that CD90 is also strongly associated with endogenous MSCs,^[^ ^45^ ^]^ further investigation was conducted using scratch and Transwell assays to determine whether the homed cells originated from endogenous sources, focusing on DFH@MSC+‐mediated stem cell migration, which is a critical mechanism in endogenous stem cell recruitment.

**Figure 6:**
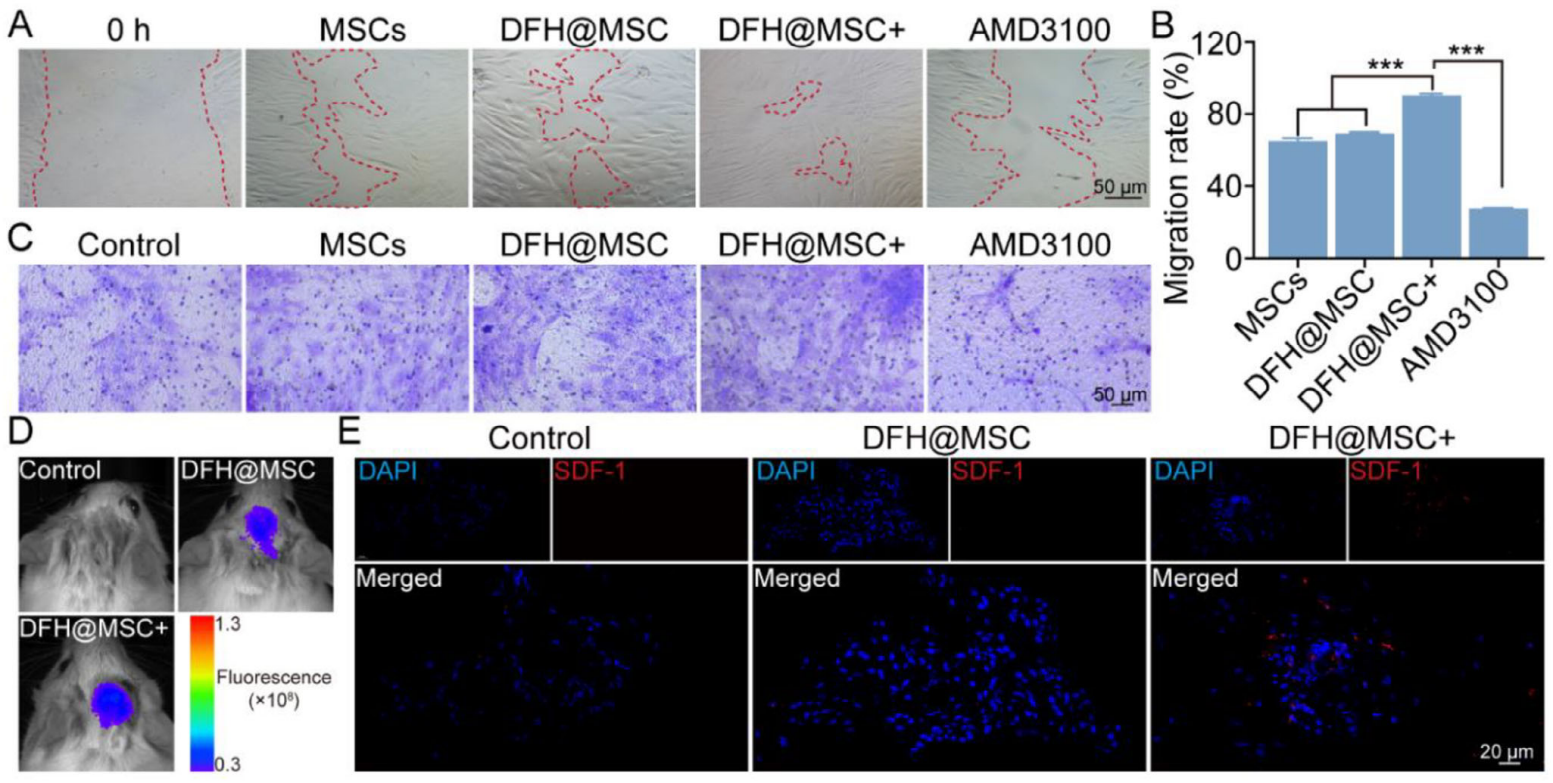
DFH@MSC+‐mediated stem cell migration in vitro and in vivo. A) Representative images of MSC migration in a scratch assay after treatment with conditioned medium. B) Quantitative analysis of the migration rate in the scratch assay. C) Representative images of MSC migration in a Transwell system after treatment with conditioned medium. D) In vivo imaging of MSC recruitment to the cranial bone defect area at 3 days post‐implantation. E) Immunofluorescence staining for SDF‐1 at 2 weeks post‐implantation.

The scratch assay revealed that photothermal stimulation significantly enhanced MSC migration in DFH@MSC+ compared to DFH@MSC (**Figure** **6**A), with quantitative analysis revealing a 1.3‐fold increase in migratory capacity (Figure 6B). This enhanced migration was further confirmed by Transwell assay, where DFH@MSC+ showed significantly more MSC migration to the lower chamber than both the DFH@MSC and normal MSCs groups (Figure 6C). To further investigate the relationship between stem cell recruitment and DFH@MSC+ in vivo, tail intravenous injection of DiR‐MSC, as a marker of peripheral stem cells, was performed three days prior to cranial bone defect construction and hydrogel implantation.^[^ ^46^ ^]^ In vivo imaging at three days post‐implantation showed significantly stronger fluorescence intensity in DFH@MSC+ compared to DFH@MSC (Figure 6D; Figure S19, Supporting Information), confirming its superior capacity for recruiting circulating stem cells in the complex immune microenvironment. Consistent with these findings, immunofluorescence staining for SDF‐1 in the skull defect further confirmed that DFH@MSC+ induced higher SDF‐1 expression than both the DFH@MSC and control groups (Figure 6E), providing additional evidence for the stem cell homing effect mediated by DFH@MSC+. Together, these results suggest that DFH@MSC+ actively promotes the recruitment of endogenous stem cells, which act as the “first responders” in tissue repair by migrating to the injured sites,^[^ ^46^, ^47^ ^]^ thereby potentiating regenerative outcomes.

### 2.5 Mechanism of DFH@MSC+‐Mediated Stem Cell Homing

**Figure 7:**
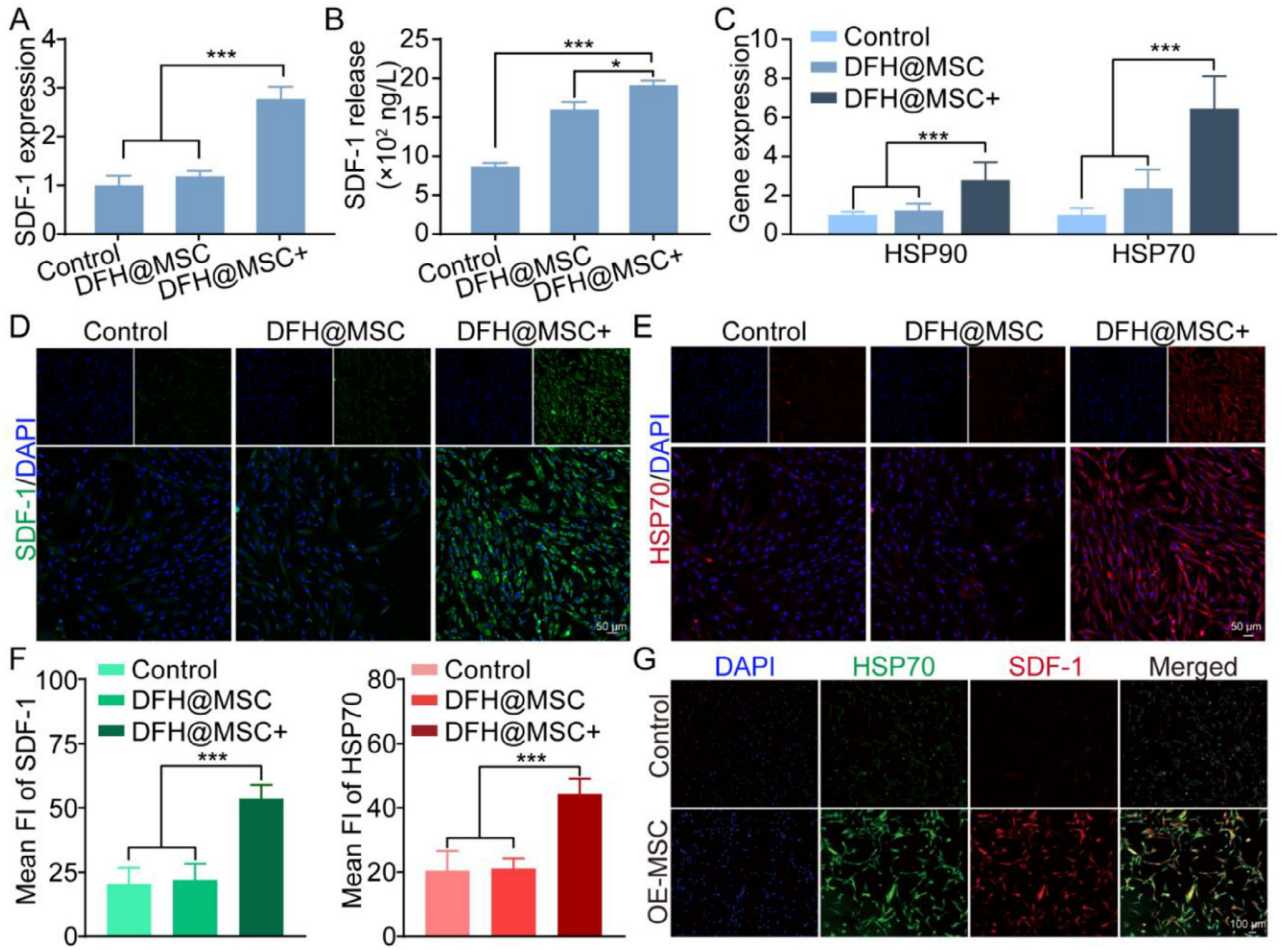
Mechanistic investigation of SDF‐1‐mediated stem cell migration. A) Relative SDF‐1 mRNA expression analyzed by qRT‐PCR (n = 5). B) SDF‐1 secretion from DFH@MSC detected by ELISA (n = 3). C) Relative expression of HSP90 and HSP70 analyzed by qRT‐PCR (n = 5). D)‐F) Immunofluorescence staining for SDF‐1 and HSP70. G) Immunofluorescence staining for SDF‐1 in HSP70‐overexpressing MSCs (OE‐MSC) and normal MSCs (Control).

To elucidate the potential mechanism by which DFH@MSC+ enhances stem cell recruitment, we systematically evaluated the expression and secretion of stromal cell‐derived factor‐1 (SDF‐1, also known as CXCL12), a key chemokine involved in stem cell homing.^[^ ^48^, ^49^, ^50^ ^]^ MSCs were cultured with DFH@MSC, with or without photothermal stimulation. qRT‐PCR analysis revealed that the SDF‐1 mRNA expression level was significantly elevated in the DFH@MSC+ group compared to the DFH@MSC and control groups (**Figure** **7**A). Consistently, ELISA showed a marked upregulation of secreted SDF‐1 in the DFH@MSC+ group (Figure 7B).

To further verify the involvement of the SDF‐1𝛼/CXCR4 axis in the migration of MSCs induced by DFH@MSC+, CXCR4 activity was blocked with AMD3100.^[^ ^46^ ^]^ While the AMD3100 treatment did not affect cell viability (Figure S20, Supporting Information), the migration assay demonstrated a significant reduction in MSC migration compared to DFH@MSC+ treatment alone (Figure 6A–C). These results collectively demonstrate that DFH@MSC+ enhances stem cell homing, with modulation of the SDF‐1/CXCR4 axis representing a significant underlying mechanism.

To elucidate the molecular mechanisms underlying the photothermal stimulation‐mediated paracrine response of MSCs in DFH, we conducted qRT‐PCR analysis to evaluate the expression of heat shock proteins (HSP70 and HSP90), which are critical regulators of cellular stress responses. The DFH@MSC+ group exhibited significant upregulation of both HSP70 and HSP90 (Figure 7C). Notably, HSP70 expression in the DFH@MSC+ group was 6.5‐fold higher after 3 days of culture compared to the group without NIR laser treatment. Immunofluorescence staining further confirmed that photothermal stimulation promoted HSP70 and SDF‐1 expression in MSCs (Figure 7D–F). To directly assess the role of HSP70 in the paracrine response, we overexpressed HSP70 in MSCs (OE‐MSC) using a lentiviral vector, which significantly increased SDF‐1 secretion (Figure 7G), highlighting HSP70 as a crucial mediator of the thermal‐induced SDF‐1 upregulation. These results suggest that HSP70 activation is a pivotal mechanism driving the paracrine response, particularly the enhanced secretion of SDF‐1 following photothermal stimulation.

## 3 Discussion and Conclusion

This study presents a dynamic fibrous hydrogel (DFH) designed to overcome critical limitations in MSC‐based cranial bone repair, specifically targeting the hostile in vivo microenvironment characterized by oxidative stress and inflammation that compromise MSC survival and paracrine function.

Although ECM‐like biomimetic or functional (e.g., photothermal and ROS‐scavenging) hydrogels have been widely reported, DFH represents a paradigm shift in regenerative hydrogel design by unifying structural biomimicry, dynamic functionality, and therapeutic intelligence. Its superiority manifests in three critical aspects: First, its innovative design achieves a breakthrough through the covalent crosslinking of nature‐derived TH (a polyphenol‐rich, fibrous material) with gelatin, creating a system that simultaneously replicates the fibrous microstructure of the native ECM while integrating photothermal responsiveness and intrinsic antioxidant properties. This contrasts with conventional approaches, which either rely on static ECM analogs or isolated functional components without synergistic integration. Second, DFH exhibits superior performance through unique functional synergy, enabling spatiotemporal control of MSC paracrine secretion via photothermal triggering, while safeguarding cell viability through its intrinsic antioxidant capacity. Furthermore, it recruits endogenous stem cells through the triggered release of SDF‐1, creating a self‐reinforcing therapeutic cascade unmatched by existing hydrogels that rely on exogenous SDF‐1 delivery, which face challenges such as high cost, short half‐life, burst release, and off‐target effects.^[^ ^51^, ^52^, ^53^, ^54^, ^55^ ^]^ Third, DFH represents a unique functional mechanism that combines microenvironment responsiveness, immunomodulation, and stem cell protection/activation into a single biomimetic platform, a substantial advancement over previous hydrogels that target only individual aspects of regeneration. By transforming passive cell delivery into active microenvironment regulation, DFH offers an intelligent, multifunctional solution for cranial bone regeneration, simultaneously addressing structural, biochemical, and cellular challenges.

DFH was rationally designed to replicate both the ECM's static cues (fibrous architecture, mechanical robustness, and biochemical functionality) and dynamic microenvironmental responsiveness, transforming passive cell delivery into active microenvironment regulation. This dual capability enhances the therapeutic benefits of MSCs in bone regeneration through three mechanisms:

1. Microenvironment protection: DFH, enriched with polyphenol‐rich TH, demonstrates strong ROS‐scavenging capacity (Figure 3A), significantly reducing intracellular ROS accumulation (Figure 3B,C) and protecting encapsulated MSCs from oxidative damage (Figure 3D–F), which represents one of the most critical challenges in MSC‐based bone tissue engineering.^[^ ^17^, ^18^, ^19^ ^]^ While the antioxidant and photothermal properties of DFH declined at two weeks (Figure S9 and S11, Supporting Information) in parallel with DFH degradation (Figure S13, Supporting Information), its early‐phase activity during the inflammatory stage is decisive for directing subsequent bone healing,^[^ ^56^ ^]^ highlighting that DFH primarily exerts its therapeutic effect when it is most needed.
2. Dynamic immunomodulation: DFH@MSC exhibits strong immunomodulatory capacity, further amplified by photothermal stimulation (Figure 5D,E). The pronounced polarization of macrophages toward the M2 phenotype in the DFH@MSC+ group highlights a shift toward a pro‐regenerative immune microenvironment,^[^ ^38^, ^43^ ^]^ indicating that DFH not only protects MSCs but also creates a more favorable immune environment for tissue repair. Although this study focuses on macrophage polarization, the broader immunomodulatory potential of DFH@MSC deserves mention. It is well‐established that MSCs can secrete TGF‐β, a cytokine implicated in various immunoregulatory processes, including the induction and/or expansion of regulatory T cells (Tregs).^[^ ^57^, ^58^, ^59^, ^60^, ^61^, ^62^ ^]^ Our data show that photothermal activation of DFH@MSC (DFH@MSC+) significantly upregulates TGF‐β expression both in vitro (Figure S21, Supporting Information) and in vivo (Figure S22, Supporting Information), suggesting a potential ancillary mechanism for indirect immune modulation. However, we emphasize that the present data do not provide functional evidence for T cell modulation in our system, which would require direct co‐culture and functional assays. Thus, the role of TGF‐β in mediating adaptive immune responses herein remains an open question for future study.
3. Active stem cell recruitment: The photothermal responsiveness of DFH activates MSC paracrine signaling, particularly upregulating SDF‐1, a key chemokine for endogenous stem cell homing, thereby initiating intrinsic repair mechanisms.^[^ ^51^, ^52^, ^53^, ^54^, ^55^ ^]^ Our prior research has demonstrated that the paracrine activity of MSCs promotes the migration and recruitment of vascular endothelial cells,^[^ ^44^ ^]^ while others have revealed that MSC‐derived SDF‐1 facilitates the migration of endothelial progenitor cells via CXCR4/phosphoinositide 3‐kinases (PI3K)/protein kinase B (AKT) signaling pathway.^[^ ^63^ ^]^ However, the potential of MSC‐secreted SDF‐1 in recruiting endogenous stem cells remains underexplored. Although 3D porous hydrogel microspheres enhanced MSC paracrine function, they did not improve stem cell migration compared to MSC‐free constructs,^[^ ^64^ ^]^ likely due to their static design, which inadequately mimics dynamic physiological conditions.^[^ ^65^ ^]^ Supporting this, recent findings demonstrated that MSCs preconditioned under a pulsed electromagnetic field secreted higher SDF‐1 levels, enhancing endogenous stem cell homing.^[^ ^66^ ^]^ Consistent with these observations, our current findings confirmed that combining a 3D fibrous structure with dynamic photothermal stimulation significantly enhances the MSC paracrine secretion of SDF‐1 (Figure 7), thereby enhancing stem cell migration and recruitment.

Critically, HSP70 activation is a key mechanism driving enhanced SDF‐1 secretion following photothermal stimulation. The advantage of this HSP70‐mediated pathway is its endogenous, cell‐driven nature. Unlike exogenous SDF‐1 delivery, which suffers from poor stability and burst release,^[^ ^55^, ^67^ ^]^ photothermal stimulation triggers a natural stress response that promotes sustained, controlled SDF‐1 secretion. This mechanism therefore provides superior spatiotemporal regulation of chemokine release, more closely mimicking physiological recruitment signals and potentially improving efficacy at the target site. Additionally, overexpression of HSP70 in MSCs significantly boosted SDF‐1 secretion (Figure 7G), reinforcing its role in the paracrine response and enhancing MSC functionality in a dynamic 3D microenvironment, ultimately boosting DFH's regenerative potential for stem cell recruitment.

For translating photothermal hydrogels into tissue regeneration therapies, the newly developed DFH addresses the key clinical translation challenges:^[^ ^68^ ^]^ sustained stability, effective tissue penetration, and manufacturing scalability. DFH demonstrates reliable in vivo photothermal stability during the first week and shows no adverse effects after implantation (Figure 4D–G), confirming both biocompatibility and long‐term safety. DFH operates reliably within the established safe thermal range (≈42 °C) through precise laser power modulation (Figure 3G–J), while the 808 nm NIR excitation minimizes tissue scattering and enhances penetration depth. Composed of naturally abundant, biocompatible gelatin and TH, and fabricated via a simplified one‐step oxidative crosslinking process at physiological temperature, DFH ensures cost‐effectiveness and scalability for industrial production. Together, these demonstrated capabilities, including controlled hyperthermia, deep tissue compatibility, and scalable manufacturing, establish DFH as a promising candidate with strong potential for successful clinical translation.

In conclusion, by integrating ECM‐mimetic static cues with dynamic photothermal/oxidative responsiveness, DFH simultaneously addresses microenvironment protection, immunomodulation, and stem cell recruitment, representing a significant advancement in bone tissue engineering. Most significantly, DFH establishes a photothermal‐enhanced “stem cell recruits stem cell” paradigm, leveraging both endogenous and exogenous MSC populations, and offering a transformative framework for efficient tissue regeneration and broader applications.

## 4 Experimental Section

### Isolation of TH

TH was separated from tea using a blender. Briefly, commodity tea products were placed in a blender for complete grinding, and then a 200‐mesh sieve was used to separate the trichomes from the powdered tea materials.^[^ ^69^ ^]^

### Total Phenolic Assay

The total phenolic content of TH was determined by the Folin‐Ciocalteu method.^[^ ^70^ ^]^ Pre‐weighed TH and GA at varying concentrations were mixed with Folin‐Ciocalteu reagent (5 mL, 10% v/v). After 6 min of incubation, sodium carbonate (4 mL, 7.5% w/v) was added. The mixture was thoroughly stirred and incubated at room temperature for 60 min before measuring the absorbance at 765 nm using a spectrophotometer. The total phenolic content in TH was expressed as (mg of GA equivalent)/(g of TH).

### Preparation of DFH

To fabricate DFH, a certain amount of TH was added to a gelatin (Aladdin, China) solution (15% w/v) and vortex‐mixed for 5 min to ensure uniform mixing. The hydrogel formulations containing varying amounts of TH (0%, 5%, 10%, and 20% weight of gelatin) were prepared and designated as Gel, DFH5, DFH10, and DFH20, respectively. Subsequently, SP was added to the mixture. The resulting precursor solution was then placed in a 37 °C incubator for gelation.

### Injection Test

The pre‐gel solution was loaded into a syringe and extruded through an 18 G needle. The extruded filaments were observed under an inverted microscope.

### Mechanical Property

The shear strength of the hydrogel was assessed using a wedge‐shaped cutting device. A universal testing machine applied controlled shear forces through a tapered blade, progressively separating the hydrogel sample until failure. The maximum shear stress recorded during the test was used to determine the hydrogel's shear strength.

### Water Absorption Experiment

The weight and length of the DFH were first recorded before soaking in deionized water at 37 °C for a set period. After removing excess moisture from the hydrogel surface, the weight and length of the swollen hydrogel were immediately recorded. Water uptake and swelling ratio were calculated using the following formula:^[^ ^71^ ^]^

(1) $$Wateruptake\left(\%\right)=\left(W_{t}-W_{0}\right)\times 100/W_{0}$$

(2) $$Swellingratio\left(\%\right)=\left(L_{t}-L_{0}\right)\times 100/L_{0}$$

where W_t_ is the weight of the sample at time t, W_0_ is the initial weight of the sample, L_t_ is the length of the sample at time t, and L_0_ is the initial length of the sample.

### Free radical Scavenging Capability

The antioxidant activity of DFH was assessed using the DPPH radical assay. Briefly, pre‐weighed DFH was added to DPPH solution (100 µM) (Macklin, China) and incubated at 37 °C in the dark. After 30 min, the absorbance of the reaction solution was measured at 517 nm using a microplate reader. The residual DPPH was calculated as: residual DPPH (%) = OD_S_×100/OD_C_, where OD_C_ represents the absorbance of the control sample (DPPH solution without DFH), and OD_S_ is the absorbance of the DFH‐treated mixture.

The ABTS^+^∙ scavenging activity of DFH was further evaluated. The ABTS^+^∙ radicals were generated by mixing ABTS with potassium persulfate in darkness for 12 h. Then, pre‐weighed DFH was added to ABTS^+^∙ solution and incubated in the dark. After 30 min, the scavenging activity was determined by measuring the absorbance decrease at 734 nm. The residual ABTS was calculated using the following equation: residual ABTS (%) = A_S_×100/A_C_, where A_C_ is the absorbance of ABTS^+^· solution without samples, and A_S_ is the absorbance of the sample particles mixed with ABTS^+^· solution.

The H_2_O_2_ scavenging capacity of DFH was tested using a commercial H_2_O_2_ colorimetric detection kit (Sangon Biotech, Shanghai, China). Pre‐weighed DFH was incubated with H_2_O_2_ (100 µM) for 30 min, followed by measuring the absorbance at 550 nm following the manufacturer's protocol.^[^ ^10^ ^]^

### Cytotoxicity Test

The hydrogels were sterilized by resuspension in 75% (v/v) ethanol, followed by three sequential washes with PBS (Keygen, Jiangsu, China). The sterilized hydrogels were incubated in serum‐free medium for 3 days at 37 °C under 5% CO_2_ to collect degradation extracts. The conditioned medium was centrifuged to remove particulates and stored at ‐20 °C until use. MSCs were seeded in 96‐well plates (1 × 10^4^ cells/well) and cultured at 37 °C for 24 h, followed by the addition of the hydrogel degradation extracts. After 24 h of incubation, the live and dead cells were stained with Calcein‐AM and PI (Keygen, Jiangsu, China), respectively. Cells cultured in fresh cell culture medium served as the control.

### Cellular ROS Scavenging Activity

MSCs (Procell, China) were cultured in DMEM+GlutaMAX medium (Gibco, USA) supplemented with 10% (v/v) FBS and 1% (w/v) penicillin/streptomycin (Yeasen, China) in a humidified atmosphere containing 5% CO_2_ at 37 °C. After reaching ≈80% confluence, the cells were incubated with H_2_O_2_ (100 µM) in the presence or absence of DFH (50 µg/mL). For live/dead cell staining, cells were stained with Calcein‐AM and PI for 15 min in the dark, followed by imaging with a fluorescence microscope. For ROS detection, cells were incubated with DCFH‐DA (Solarbio, Beijing, China) for 20 min in the dark, followed by imaging with a fluorescence microscope.

### In Vivo Retention and Survival of MSCs In Vivo

To track the cell survival, MSCs were transduced with lentivirus particles expressing red fluorescent protein (RFP‐MSCs). For in vivo transplantation, hydrogel samples (50 µL total with 5 × 10^5^ cells) were injected subcutaneously into the scalp of ICR mice. RFP‐MSCs resuspended at the same concentration in saline (50 µL) were also injected as controls. To monitor cell viability and distribution, fluorescence imaging was performed on days 0, 1, 3, and 7 post‐implantation using an IVIS Spectrum system.^[^ ^72^ ^]^ Fluorescence intensity (FI) was quantified using Living Image software by measuring total FI in standardized ROIs, subtracting background, and normalizing to baseline (0 h).

### In Vivo Recruitment Assay

Three days before cranial bone defect construction and DFH@MSC implantation, mice in each group were injected with DiR‐labeled MSCs (DiR‐MSC) via tail vein injection. Three days post‐implantation, fluorescence imaging was performed using an IVIS Spectrum system. FI was quantified using Living Image software by measuring total FI in standardized ROIs, subtracting background.

### Photothermal Properties

The photothermal performance of the hydrogel was evaluated by exposing it to an 808 nm NIR laser with different power densities (0.28, 0.32, and 0.40 W cm^−2^). The temperatures of the samples under various irradiations were recorded using an infrared thermal camera. To assess cyclic stability, the hydrogel was subjected to five consecutive cycles of laser irradiation (1 min per cycle at 0.38 W cm^−2^), followed by cooling to room temperature. For sustained stability, the hydrogel was irradiated at 0.50 W cm^−2^ to rapidly increase the temperature to 42 °C, after which the power density was reduced to 0.32 W cm^−2^ to maintain a stable temperature of 42 °C for 10 min.

### Hemolytic Activity

Murine red blood cell suspension in PBS was added to the tubes containing serial concentrations of DFH. After incubation for 1 h, the tubes were centrifuged at 1200 g for 5 min, and the supernatant was collected and photographed, followed by measuring the optical density at 490 nm using a microplate reader. 1% Triton X‐100 and PBS were used as the positive and negative controls, respectively.

### Animal Experiments

ICR mice (6‐8 weeks old) were purchased from the Laboratory Animal Center of Yangzhou University. All animal experiments were conducted in accordance with the guidelines of the Institutional Animal Care and Use Committee of the Yangzhou University (SYXK(Su) 2022‐0044). A full‐thickness cranial bone defect (3 mm in diameter) was created on the mouse's calvaria using a 1 mm diameter cranial drill with continuous saline irrigation to reduce local temperature during drilling. The following hydrogel was implanted: DFH (with and without photothermal stimulation), DFH@MSC (with and without photothermal stimulation), and free MSCs at the injury sites. After 2 weeks, the bone tissues were collected and fixed with 4% paraformaldehyde and scanned using a micro‐CT scanner (Servicebio, Wuhan, China). The region of interest (ROI) with a diameter of 3 mm was defined to evaluate the level of bone regeneration.

### H&E and Masson's Trichrome Staining

At predetermined time points, defective bone tissues were harvested and processed for histological analysis. The specimens were fixed with 4% paraformaldehyde for 1 week, decalcified in 10% EDTA, and then dehydrated through a graded ethanol series. After embedding in paraffin, the specimens were cut into slices (≈5 µm) using a rotary microtome, followed by deparaffinization and staining with H&E and Masson's trichrome. Sections were then observed and imaged under a light microscope.

Tissue sections were stained with mouse anti‐CD90, anti‐iNOS, anti‐CD206, anti‐SDF‐1, and anti‐TGF‐β antibodies, followed by appropriate secondary antibodies. Nuclei were stained with DAPI, and fluorescence images were acquired using a fluorescence microscope.

### Preparation of Conditioned Media

MSCs were cultured in three groups, including TCP (tissue culture plastic), DFH@MSC, and DFH@MSC+. For DFH@MSC+ group, the cell‐seeded hydrogels were irradiated with 808 nm NIR light and heated to ≈42 °C for 10 min/day. After 72 h of culture at 37 °C, the conditioned media from all groups were collected, followed by centrifugation at 3000 rpm for 10 min to remove any dead cells and cellular debris.

### ELISA

SDF‐1 in cell supernatants was measured using ELISA kits according to the manufacturer's instructions.

### qRT‐PCR Assay

MSCs at a density of 1×10^5^ cells per well were co‐cultured with DFH@MSC in 6‐well plates for 72 h at 37 °C. Total RNA was extracted using an RNA extraction kit (New Cell & Molecular Biotech, Suzhou, China). Complementary DNA (cDNA) was synthesized by NovoNGS Second‐Strand cDNA Synthesis Kit (Novoprotein, Shanghai, China, N422) with total RNA and random primers. Subsequently, qRT‐PCR was conducted using SYBR Green qPCR Master Mix (YIFEIXUE BioTech, Nanjing, China).

### Scratch Test

When MSCs reached 90% confluence, a scratch wound was generated in the cell monolayer using a sterile plastic 200 µL micropipette tip. After washing twice with PBS, the conditioned medium was added to the plates. After 24 h of incubation, the cells were visualized under a light microscope. The migration ratio was quantified using Image Pro Plus software.

### Transwell Assay

MSCs were cultured in the upper chamber while conditioned medium was placed in the lower chamber and incubated for 24 h. Cells were stained with crystal violet and imaged under a light microscope. The cell migration and invasion stain kit was purchased from Beijing Solarbio Science & Technology Co., Ltd. (Beijing, China). To investigate the signaling pathways, MSCs were pre‐co‐cultured with CXCR4 inhibitor AMD3100 (10 µmol L^−1^) for 24 h^[^ ^46^ ^]^ and then employed for the scratch test and Transwell assay.

### Immunofluorescence Staining

MSCs were co‐cultured with DFH@MSC+ in 24‐well plates for 72 h at 37 °C. The cells were then washed three times with PBS, fixed with 4% paraformaldehyde, permeabilized with 0.1% Triton X‐100 for 30 min, and blocked with 5% bovine serum albumin (BSA) for 1 h. The cells were incubated with anti‐SDF‐1 antibody at 4 °C overnight, and subsequently stained with a goat anti‐mouse secondary antibody for 2 h at room temperature. Nuclei were counterstained with DAPI. Immunofluorescence staining images were captured under a fluorescence microscope.

### HSP70 Over‐Expressed MSCs (OE‐MSC)

To generate HSP70‐overexpressing MSCs (OE‐MSC), the HSP70‐lentiviral vector was used to infect MSCs culture on DFH at a MOI of 50, and the transfected cells were selected with 2 µg mL^−1^ puromycin for 7 days. For immunofluorescence analysis, both OE‐MSC and normal MSCs were fixed with 4% paraformaldehyde, permeabilized with 0.1% Triton X‐100, and blocked with 5% BSA. Cells were then stained with primary antibodies against HSP70 (1:200) and SDF‐1 (1:100) overnight at 4 °C, followed by incubation with appropriate fluorescent secondary antibodies (1:500) for 1 h at room temperature. Nuclei were counterstained with DAPI, and images were acquired using a confocal microscope.

### Statistical Analysis

Data are presented as mean±standard deviation (SD). Experimental data were analyzed using GraphPad Prism software. Two independent sample t‐test was performed for comparisons between two groups, while the one‐way analysis of variance (ANOVA) was used for comparisons among multiple groups. A value of p<0.05 was considered statistically significant (*ns*: no significant, ^*^ *p*< 0.05, ^**^ *p*< 0.01, and ^***^ *p*< 0.001).

## Conflict of Interest

The authors declare no conflict of interest.

## Supporting information



Supporting Information

Supplementary Video 1

Supplementary Video 2

Supplementary Video 3

Supplementary Video 4

Supplementary Video 5

## Data Availability

The data that support the findings of this study are available in the supplementary material of this article.

## References

[1] A.‐M. Wu , C. Bisignano , S. L. James , G. G. Abady , A. Abedi , E. Abu‐Gharbieh , R. K. Alhassan , V. Alipour , J. Arabloo , M. Asaad , W. N. Asmare , A. F. Awedew , M. Banach , S. K. Banerjee , A. Bijani , T. T. M. Birhanu , S. R. Bolla , L. A. Cámera , J.‐C. Chang , D. Y. Cho , M. T. Chung , R. A. S. Couto , X. Dai , L. Dandona , R. Dandona , F. Farzadfar , I. Filip , F. Fischer , A. A. Fomenkov , T. K. Gill , Lancet 2021, 2, 580.

[2] Z. Cao , Y. Bian , T. Hu , Y. Yang , Z. Cui , T. Wang , S. Yang , X. Weng , R. Liang , C. Tan , J. Materiomics 2023, 9, 930.

[3] L. Zhou , H. Liu , B. Zhang , C. Wei , S. Zhou , X. Huang , X. Zhong , L. Zhang , W. Bi , J. Liu , Y. Liang , L. Jin , R. Guo , Adv. Funct. Mater. 2024, 34, 2314330.

[4] G. Lu , Y. Xu , Q. Liu , M. Chen , H. Sun , P. Wang , X. Li , Y. Wang , X. Li , X. Hui , E. Luo , J. Liu , Q. Jiang , J. Liang , Y. Fan , Y. Sun , X. Zhang , Nat. Commun. 2022, 13, 2499. 35523800 10.1038/s41467-022-30243-5 PMC9076642

[5] X. Zhang , B. Yang , L. Feng , X. Xu , C. Wang , Y.‐w. Lee , M. Wang , X. Lu , L. Qin , S. Lin , L. Bian , G. Li , Bioact. Mater. 2024, 41, 440. 39188381 10.1016/j.bioactmat.2024.07.036 PMC11347042

[6] F. Shang , Y. Yu , S. Liu , L. Ming , Y. Zhang , Z. Zhou , J. Zhao , Y. Jin , Bioact. Mater. 2021, 6, 666. 33005830 10.1016/j.bioactmat.2020.08.014 PMC7509590

[7] H.‐J. Park , H. Hong , R. Thangam , M.‐G. Song , J.‐E. Kim , E.‐H. Jo , Y.‐J. Jang , W.‐H. Choi , M.‐Y. Lee , H. Kang , K.‐B. Lee , Nanomaterials 2022, 12, 1377. 35458085 10.3390/nano12081377 PMC9028203

[8] R. O. Hynes , Science 2009, 326, 1216. 19965464 10.1126/science.1176009 PMC3536535

[9] Q. Yang , Y. Miao , J. Luo , M.‐G. Song , J.‐E. Kim , E.‐H. Jo , Y.‐J. Jang , W.‐H. Choi , M.‐Y. Lee , H. Kang , K.‐B. Lee , ACS Nano 2025, 19, 6613.39995326

[10] J. Chen , C. Pan , Y. Gao , Q. Chen , X. An , Z. Liu , ACS Appl. Mater. Interfaces 2024, 16, 17120. 38554083 10.1021/acsami.3c18284

[11] H. Cao , L. Duan , Y. Zhang , J. Cao , K. Zhang , Signal Transduct. Tar. Ther. 2021, 6, 426. 10.1038/s41392-021-00830-x PMC8674418 34916490

[12] A. M. Rosales , K. S. Anseth , Nat. Rev. Mater. 2016, 1, 15012. 29214058 10.1038/natrevmats.2015.12 PMC5714327

[13] E. Prince , E. Kumacheva , Nat. Rev. Mater. 2019, 4, 99.

[14] W. Luo , L. Ren , B. Hu , H. Zhang , Z. Yang , L. Jin , D. Zhang , Adv. Sci. 2024, 12, 2408657. 10.1002/advs.202408657 PMC11714238 39530645

[15] X. Xie , Z. Li , X. Yang , B. Yang , Z. Zong , X. Wang , L. Duan , S. Lin , G. Li , L. Bian , J. Am. Chem. Soc. 2023, 145, 15218. 37428960 10.1021/jacs.3c02210

[16] Y. Z. Sun , R. W. Sheng , Z. C. Cao , C. Q. Liu , J. X. Li , P. Zhang , Y. Du , Q. Y. Mo , Q. Q. Yao , J. L. Chen , W. Zhang , Sci. Adv. 2024, 10, adm7164. 10.1126/sciadv.adm7164 PMC11042749 38657071

[17] S. Shen , R. Liu , C. Song , T. Shen , Y. Zhou , J. Guo , B. Kong , Q. Jiang , Nano Res. 2023, 16, 7383.

[18] K. Zhang , C. Zhang , H. Zhou , Y. Yang , Y. Wen , X. Jiao , M. Yao , Y. Wen , ACS Appl. Mater. Interfaces 2024, 16, 41869. 39101935 10.1021/acsami.4c07369

[19] O. Levy , R. Kuai , E. M. J. Siren , D. Bhere , Y. Milton , N. Nissar , M. De Biasio , M. Heinelt , B. Reeve , R. Abdi , M. Alturki , M. Fallatah , A. Almalik , A. H. Alhasan , K. Shah , J. M. Karp , Sci. Adv. 2020, 6, aba6884. 10.1126/sciadv.aba6884 PMC7439491 32832666

[20] H. Zhou , W. Zhou , X. Yao , Q. Zhao , L. Lu , Int. J. Mol. Sci. 2023, 24, 5207. 36982281 10.3390/ijms24065207 PMC10049225

[21] H. Liu , X. Qu , H. Tan , J. Song , M. Lei , E. Kim , G. F. Payne , C. Liu , Acta Biomater. 2019, 88, 181. 30818052 10.1016/j.actbio.2019.02.032

[22] X. Yu , J. Li , M. Yang , C. Chen , S. Munir , J. You , T. Yin , R. Liu , S. Xiong , Y. Hu , Food Chem. 2021, 360, 130068. 34029925 10.1016/j.foodchem.2021.130068

[23] J. Chen , X. An , L. Xu , Y. Gao , M. Zhou , Z. Liu , Small 2024, 20, 2306598. 10.1002/smll.202306598 38295133

[24] C. Huangfu , Z. Liu , X. Lu , Q. Liu , T. Wei , Z. Fan , Energy Storage Mater. 2021, 43, 120.

[25] X. Kang , P. Guan , C. Xiao , C. Liu , Y. Guan , Y. Lin , Y. Tian , K. Ren , Y. Huang , R. Fu , C. Ning , L. Fan , G. Tan , L. Zhou , Adv. Healthc. Mater. 2023, 12, 2203306. 10.1002/adhm.202203306 36708290

[26] P. Tang , T. Zheng , C. Yang , G. Li , Food Chem. 2022, 393, 133353. 35679702 10.1016/j.foodchem.2022.133353

[27] Z. Y. Qin , Y. J. Huang , S. Y. Xiao , H. Y. Zhang , Y. L. Lu , K. J. Xu , Int. J. Mol. Sci. 2022, 23, 9482.36012746

[28] J. Yang , M. Li , Y. Wang , H. Wu , T. Zhen , L. Xiong , Q. Sun , Biomacromolecules 2019, 20, 801. 30608151 10.1021/acs.biomac.8b01420

[29] X. Zhao , M. Zhang , B. Guo , P. X. Ma , J. Mater. Chem. B 2016, 4, 6644. 32263519 10.1039/c6tb01776b

[30] W. Jin , W. Xu , H. Ge , Y. Li , B. Li , RSC Adv. 2015, 5, 26496.

[31] Y. Zhao , Z. Sun. International Journal of Food Properties 2018, 20, S2822.

[32] Y. Zou , A. Zhang , L. Lin , S. A. El‐Sohaimy , Y. Li , L. Wu , H. Zhang , Int. J. Biol. Macromol. 2023, 224, 667. 36280172 10.1016/j.ijbiomac.2022.10.155

[33] X. Wu , L. Sun , J. Chen , M. Su , Z. Liu , Gels 2025, 11, 797. 41149402 10.3390/gels11100797 PMC12562426

[34] Z. Chang , S. Zhang , F. Li , Z. Wang , J. Li , C. Xia , Y. Yu , L. Cai , Z. Huang , Chem. Eng. J. 2021, 404, 126505.

[35] K. Wu , Y. Li , H. Wang , J. Xiao , W. Ma , L. Li , Food Hydrocolloid 2025, 168, 111483.

[36] S. Guo , Y. Ren , R. Chang , Y. He , D. Zhang , F. Guan , M. Yao , ACS Appl. Mater. Interfaces 2022, 14, 34455. 35857973 10.1021/acsami.2c08870

[37] W. Zhou , F. Han , C. Li , Y. Xu , Materials Technology 2022, 37, 2874.

[38] M. Wu , H. Liu , Y. Zhu , F. Chen , Z. Chen , L. Guo , P. Wu , G. Li , C. Zhang , R. Wei , L. Cai , Small 2023, 19, 2300111. 10.1002/smll.202300111 37191242

[39] W. Feng , Z. Wang , Adv. Sci. 2023, 10, 2303326. 10.1002/advs.202303326 PMC10558674 37544909

[40] S. Fang , Q. Chang , H. Jia , S. Liu , X. Deng , Y. Xie , ACS Appl. Nano Mater. 2024, 7, 13590.

[41] L. Tan , Y. Hu , M. Li , Y. Zhang , C. Xue , M. Chen , Z. Luo , K. Cai , Chem. Eng. J. 2022, 431, 133382.

[42] M. Á. Brennan , P. Layrolle , D. J. Mooney , Adv. Funct. Mater. 2020, 30, 1909125. 32952493 10.1002/adfm.201909125 PMC7494127

[43] F. L. Wei , Y. Zhai , T. F. Wang , Z. Tang , K. Shen , H. Wu , R. Zheng , M. R. Du , W. Heng , X. X. Li , X. D. Yan , Q. Y. Gao , Z. Guo , J. X. Qian , C. P. Zhou , Sci. Adv. 2024, 10, adq6700. 10.1126/sciadv.adq6700 PMC11529719 39485837

[44] J. Chen , X. Wu , Y. Zhang , Y. Xu , H. Ge , X. Ning , Nano Lett. 2022, 22, 5723. 35787105 10.1021/acs.nanolett.2c00760

[45] Z. Chen , Z. Lv , Y. Zhuang , Q. Saiding , W. Yang , W. Xiong , Z. Zhang , H. Chen , W. Cui , Y. Zhang , Adv. Mater. 2023, 35, 2300180. 10.1002/adma.202300180 37230467

[46] Z. Wang , A. Lao , X. Huang , Y. Zhou , S. G. Shen , D. Lin , Adv. Funct. Mater. 2024, 34, 2316675.

[47] H. Xia , X. Li , W. Gao , X. Fu , R. H. Fang , L. Zhang , K. Zhang , Nat. Rev. Mater. 2018, 3, 174.

[48] X. Zhang , Q. Li , L. Li , J. Ouyang , T. Wang , J. Chen , X. Hu , Y. Ao , D. Qin , L. Zhang , J. Xue , J. Cheng , W. Tao , ACS Nano 2023, 17, 6466. 36996420 10.1021/acsnano.2c11486

[49] G. Cheng , X. Wang , F. Zhang , K. Wang , Y. Li , T. Guo , N. Xu , W. Wei , S. Yan , Biomed. Pharmacother. 2024, 181, 117649. 39536539 10.1016/j.biopha.2024.117649

[50] Z. Sun , C. Lin , K. Wu , M. Wang , F. Cai , Q. Lin , M. Zhou , H. Liu , F. Yan , Mater. Des. 2021, 208, 109884.

[51] I. Safina , M. C. Embree , Acta Biomater. 2022, 143, 26. 35292413 10.1016/j.actbio.2022.03.014 PMC9035107

[52] H. Zeng , Z. Chen , P. Wei , H. Huang , B. Liu , Z. Fan , Adv. Funct. Mater. 2024, 34, 2400608.

[53] L. Luo , Y. Li , Z. Bao , D. Zhu , G. Chen , W. Li , Y. Xiao , Z. Wang , Y. Zhang , H. Liu , Y. Chen , Y. Liao , K. Cheng , Z. Li , Adv. Mater. 2023, 36, 2302686. 10.1002/adma.202302686 37665792

[54] Y. He , F. Li , P. Jiang , F. Cai , Q. Lin , M. Zhou , H. Liu , F. Yan , Bioact. Mater. 2023, 21, 223. 36157244 10.1016/j.bioactmat.2022.08.012 PMC9465026

[55] L. Xin , X. Zheng , J. Chen , S. Hu , Y. Luo , Q. Ge , X. Jin , L. Ma , S. Zhang , Adv. Healthcare Mater. 2022, 11, 2201680. 10.1002/adhm.202201680 36049781

[56] Y. Dang , Y. Zhang , G. Luo , D. Li , Y. Ma , Y. Xiao , L. Xiao , X. Wang , Appl. Mater. Today 2024, 38, 102236.

[57] V. de Araújo Farias , A. B. Carrillo‐Gálvez , F. Martín , P. Anderson , Cytokine Growth F. R. 2018, 43, 25. 10.1016/j.cytogfr.2018.06.002 29954665

[58] L. Gao , S. Cen , P. Wang , Z. Xie , Z. Liu , W. Deng , H. Su , X. Wu , S. Wang , J. Li , Y. Ouyang , Y. Wu , H. Shen , Stem Cells Transl. Med. 2016, 5, 1496. 27400793 10.5966/sctm.2015-0420 PMC5070508

[59] T. Song , A. Eirin , X. Zhu , Y. Zhao , J. D. Krier , H. Tang , K. L. Jordan , J. R. Woollard , T. Taner , A. Lerman , L. O. Lerman , Hypertension 2020, 75, 1223. 32223383 10.1161/HYPERTENSIONAHA.119.14546 PMC7219723

[60] W. Chen , Annu. Rev. Immunol. 2023, 41, 483. 36750317 10.1146/annurev-immunol-101921-045939 PMC12453633

[61] K. Lynch , O. Treacy , X. Chen , N. Murphy , P. Lohan , M. N. Islam , E. Donohoe , M. D. Griffin , L. Watson , S. McLoughlin , G. O'Malley , A. E. Ryan , T. Ritter , Mol. Ther. 2020, 28, 2023. 32531237 10.1016/j.ymthe.2020.05.023 PMC7474271

[62] M. Di Nicola , C. Carlo‐Stella , M. Magni , M. Milanesi , P. D. Longoni , P. Matteucci , S. Grisanti , A. M. Gianni , Blood 2002, 99, 3838. 11986244 10.1182/blood.v99.10.3838

[63] X. Wang , H. Jiang , L. Guo , S. Wang , W. Cheng , L. Wan , Z. Zhang , L. Xing , Q. Zhou , X. Yang , H. Han , X. Chen , X. Wu , J. Mol. Histol. 2021, 52, 1155. 34642827 10.1007/s10735-021-10008-y

[64] X. Li , X. Li , J. Yang , J. Lin , Y. Zhu , X. Xu , W. Cui , Small 2023, 19, 2207211. 10.1002/smll.202207211 36651038

[65] C. J. Derrick , E. S. Noel , Development 2021, 148, dev191320. 33674261 10.1242/dev.191320

[66] Y. Li , L. Li , M. Wang , B. Yang , B. Huang , S. Bai , X. Zhang , N. Hou , H. Wang , Z. Yang , C. Tang , Y. Li , W. Yuk‐Wai Lee , L. Feng , M. D. Tortorella , G. Li , Bioact. Mater. 2023, 28, 255. 37303853 10.1016/j.bioactmat.2023.05.003 PMC10247879

[67] R. Molina‐Peña , M. Haji Mansor , M. Najberg , J.‐M. Thomassin , B. Gueza , C. Alvarez‐Lorenzo , E. Garcion , C. Jérôme , F. Boury , Int. J. Pharm. 2021, 610, 121205. 34670119 10.1016/j.ijpharm.2021.121205

[68] S. Sun , G. Jiang , J. Dong , X. Xie , J. Liao , Y. Tian , Front. Bioeng. Biotech. 2024, 12, 1389327. 10.3389/fbioe.2024.1389327 PMC11007110 38605983

[69] P. Li , Y. Xu , Y. Zhang , J. Fu , S. Yu , H. Guo , Z. Chen , C. Chen , X. Yang , S. Wang , J. Zhao , J. Agric. Food Chem. 2020, 68, 11389. 32852206 10.1021/acs.jafc.0c04075

[70] I. Dominguez‐López , M. Pérez , R. M. Lamuela‐Raventós , Crit. Rev. Food Sci. Nutr. 2023, 64, 10048. 37283051 10.1080/10408398.2023.2220031 PMC10700652

[71] M. Farhadpour , G. Liu , Q. Zhao , Q. You , M. Pan , R. Bagheri , G. Pircheraghi , M. Shao , Chem. Eng. J. 2025, 509, 161203.

[72] L. Cai , R. E. Dewi , S. C. Heilshorn , Adv. Funct. Mater. 2015, 25, 1344. 26273242 10.1002/adfm.201403631 PMC4529129

