# Supplementary material for: Dynamic Fibrous Hydrogels for Stem Cell Homing and In Situ Bone Regeneration
Source: Adv Sci (Weinh). 2025 Nov 16;13(5):e08803. doi: 10.1002/advs.202508803 (PMC12850137; doi:10.1002/advs.202508803)
Supplement: Supplementary file 1 — Supporting Information [file ADVS-13-e08803-s001.pdf]

Supporting Information

**Dynamic fibrous hydrogels for stem cell homing and *in situ* bone regeneration**

*Jianmei Chen*, Meiling Su, Xinyu Wu, Hongyu Wu, Haotian Wu, Pinghu Zhang, Xueying An* and Zongguang Liu**

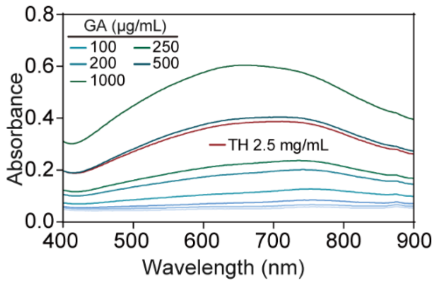


**Figure S1.** Quantification of polyphenol content in TH using the Folin-Ciocalteu assay. The absorption curves of TH (2.5 mg/mL) and gallic acid (GA) at different concentration.

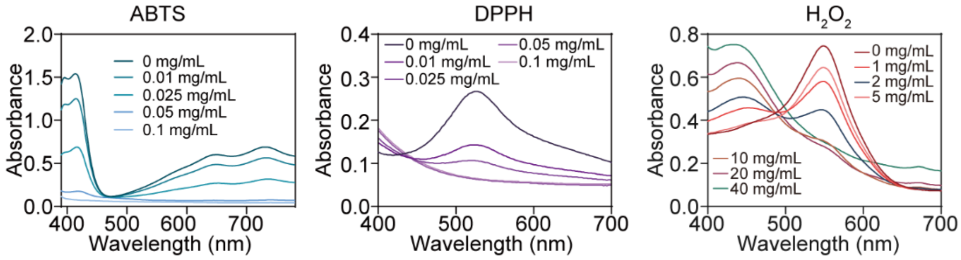


**Figure S2.** Free radical scavenging capability of TH against ABTS+·, DPPH· and $H_2O_2$. The absorption curves of (A) ABTS, (B) DPPH, and (C) $H_2O_2$ solutions treated with various concentrations of TH.

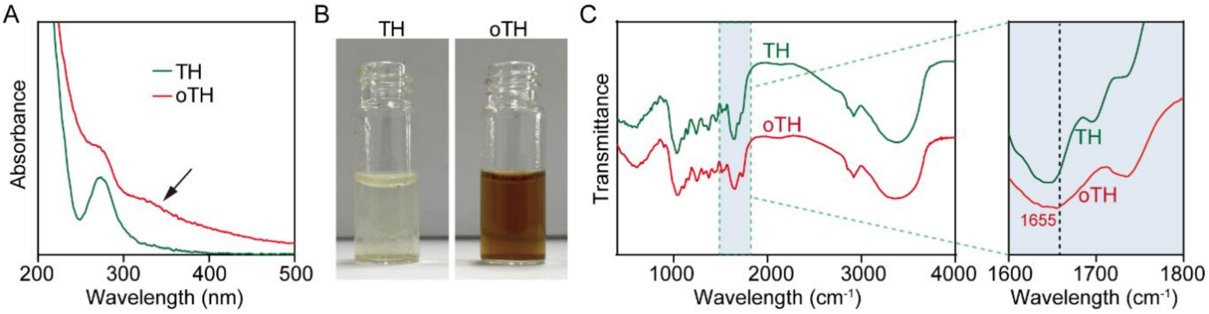


**Figure S3.** (A) Ultraviolet-visible-near-infrared (UV-Vis-NIR) absorption spectra of TH and oxidized TH (oTH). (B) Color variation between TH and oTH. (C) FTIR spectra of TH and oTH.

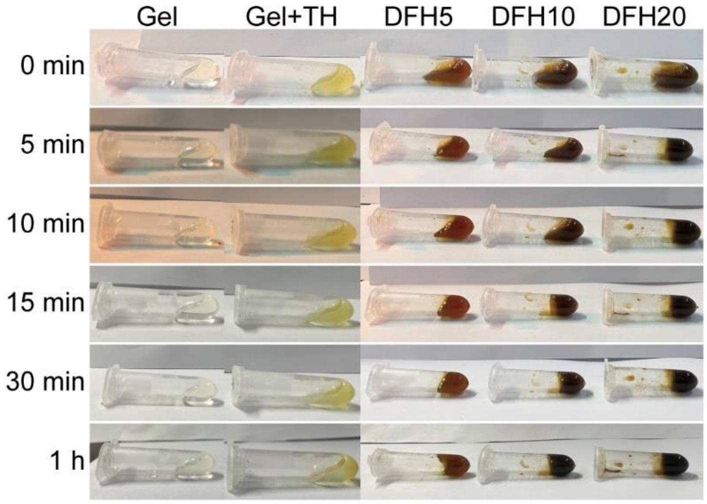


**Figure S4.** Detailed gelation process of DFH. Gel+TH: gelatin and TH mixture without SP.

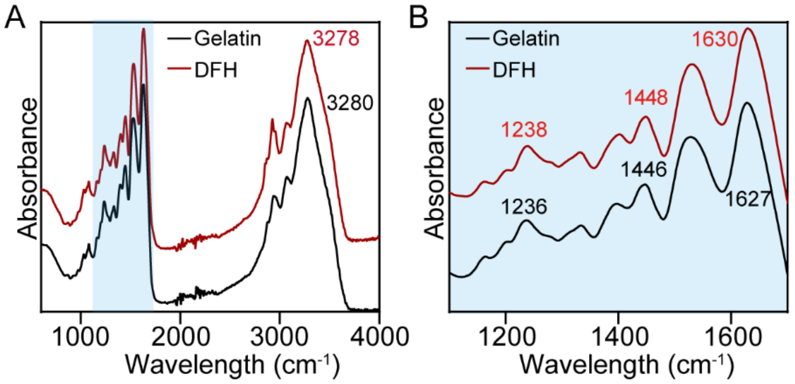


**Figure S5.** FTIR spectra of gelatin and DFH.

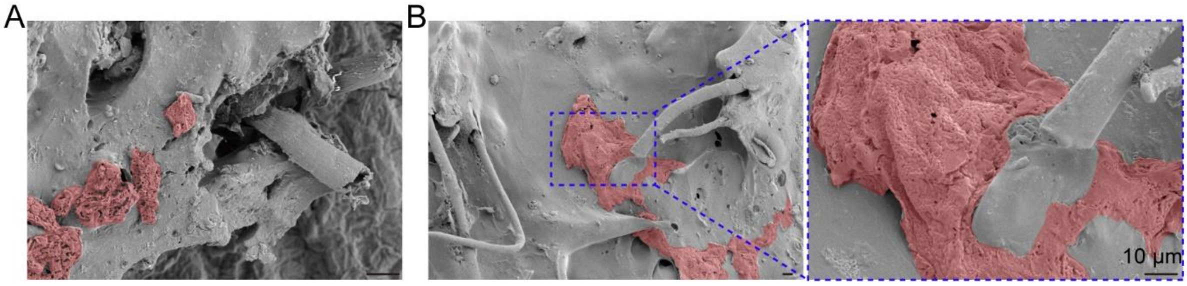


**Figure S6.** SEM images of DFH@MSC (A) immediately post-encapsulation and (B) after 3-day culture.

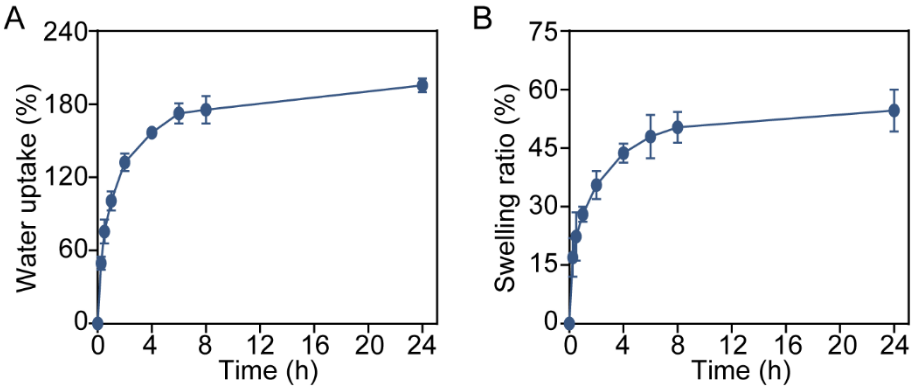


**Figure S7.** (A) Water uptake and (B) swelling ratio of DFH in PBS at 37°C.

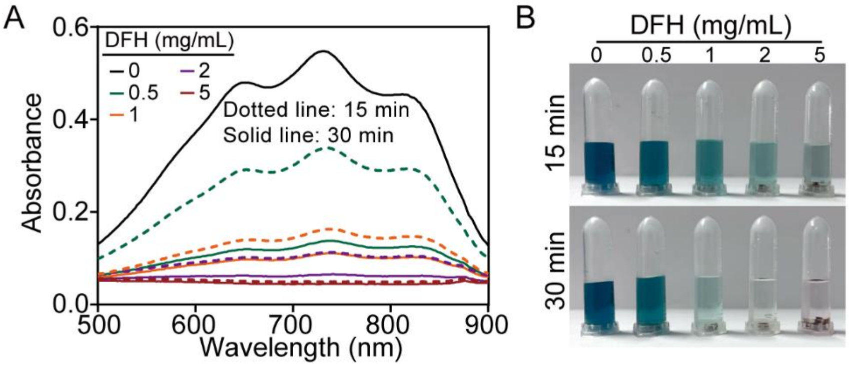


**Figure S8.** ABTS$^{+}\cdot$ scavenging capability of DFH at varying concentrations. (A) Absorption curves of ABTS solution with different concentrations of DFH. (B) Decolorization of ABTS solution by DFH.

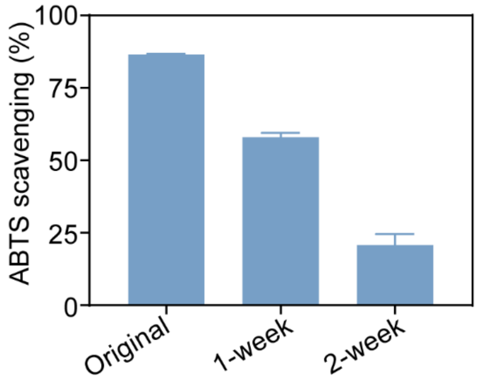


**Figure S9.** ABTS scavenging capability of DFH before and after 2-week subcutaneous implantation in mice (n=5).

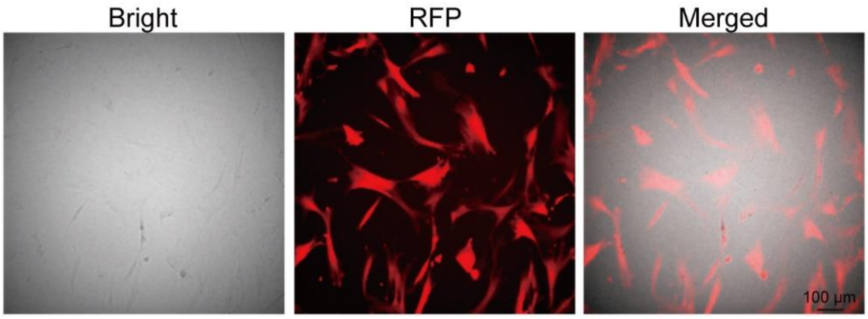


**Figure S10.** Lentivirus-mediated transfection of red fluorescent protein (RFP) gene into MSCs, establishing a stable RFP-expressing cell line (RFP-MSCs).

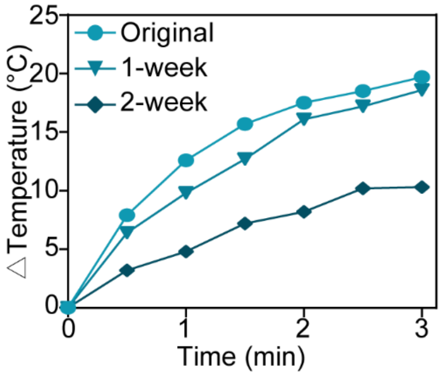


**Figure S11.** Photothermal performance of DFH before and after 2-week subcutaneous implantation in mice.

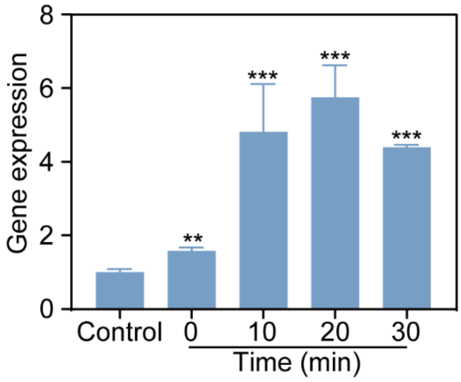


**Figure S12.** Relative VEGF expression of MSCs in DFH@MSC under varying durations of photothermal stimulation by qRT-PCR. Normal cultured MSCs served as the control (n=3).

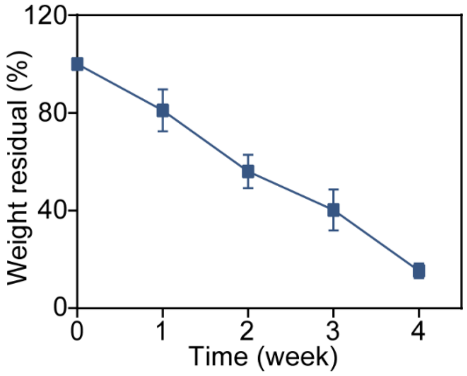


**Figure S13.** *In vivo* degradation kinetics of DFH after subcutaneous implantation in mice (n=5).

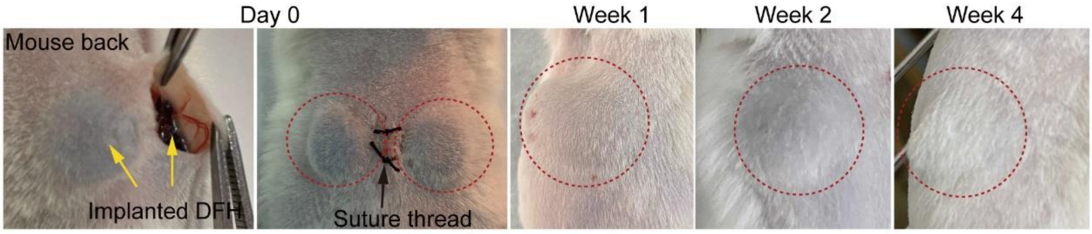


**Figure S14.** Gross morphological images of implanted DFH during 4-week subcutaneous implantation.

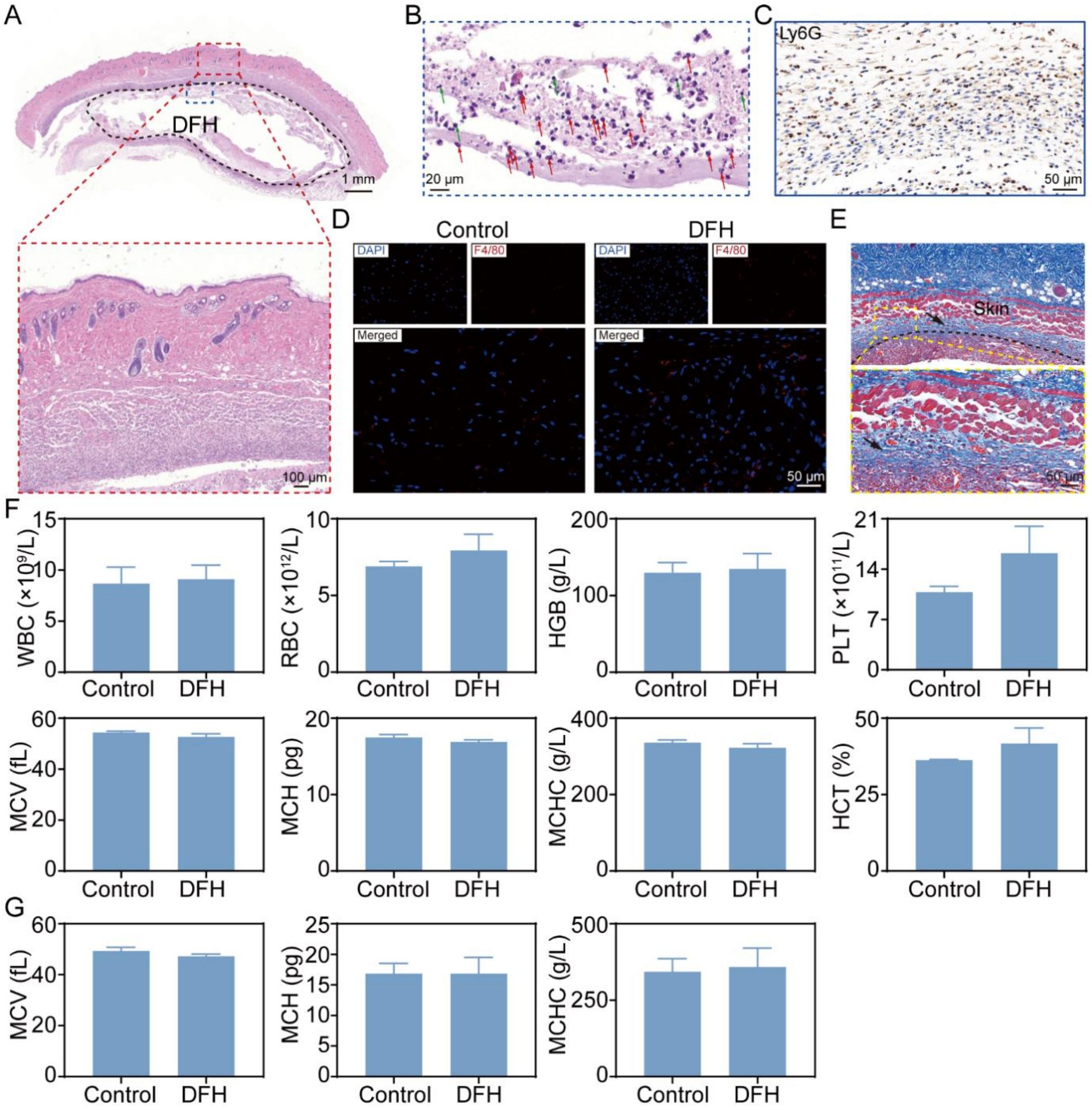


**Figure S15.** Biocompatibility of DFH in a murine subcutaneous model. (A)-(B) H&E staining at 1-week post-implantation. Black dashed lines mark the margin of implanted DFH. Red arrows indicate neutrophils. Green arrows indicate lymphocytes at 1 week post-implantation. (C) Ly6G immunofluorescence staining at 1 week post-implantation. (D) F4/80 immunofluorescence staining at 1-week post-implantation. (E) Masson staining at 1-week post-implantation. Black dashed lines mark the margin of implanted DFH. Black arrows indicate fibrous capsule. (F) Blood routine analysis at 1 week post-implantation (n=3). (G) Blood routine analysis at 4 weeks post-implantation (n=3).

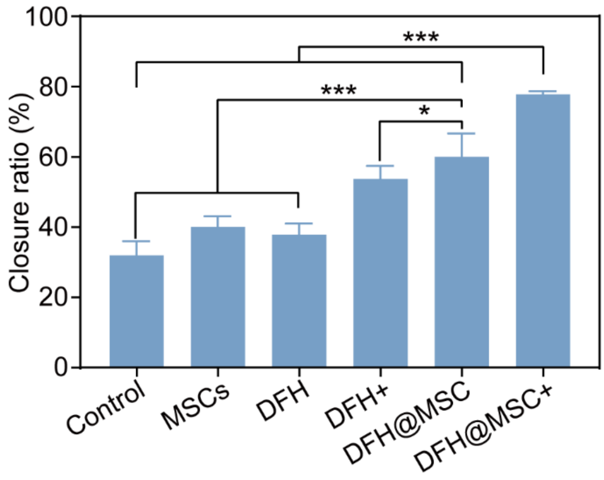


**Figure S16.** Quantitative analysis of new bone formation area across experimental groups (n=3).

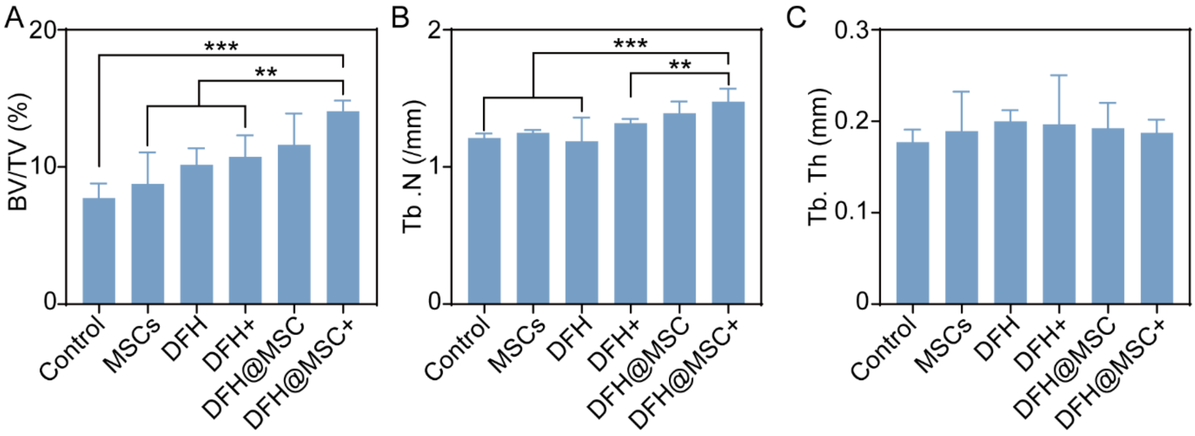


**Figure S17.** Quantitative analysis of micro-CT parameters of regenerated bone in the defect area (n=3). (A) BV/TV (bone volume/tissue volume). (B) Tb. N (trabecular number). (C) Tb. Th (trabecular thickness).

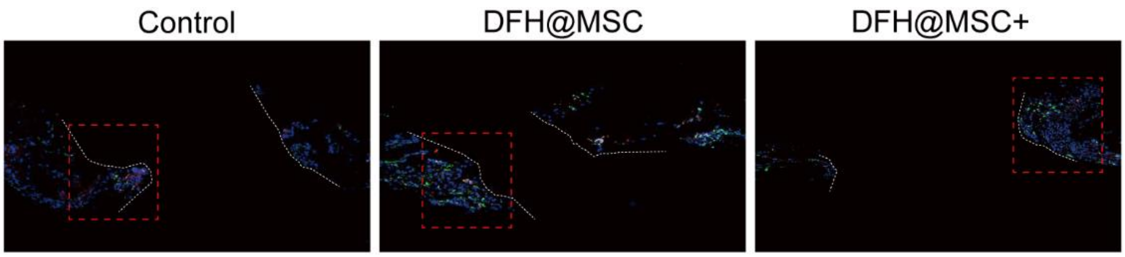


**Figure S18.** Immunofluorescence staining images of iNOS and CD206 (5× magnification). White dotted lines: bone defect margin; red dashed boxes: regions magnified in Figure 5D.

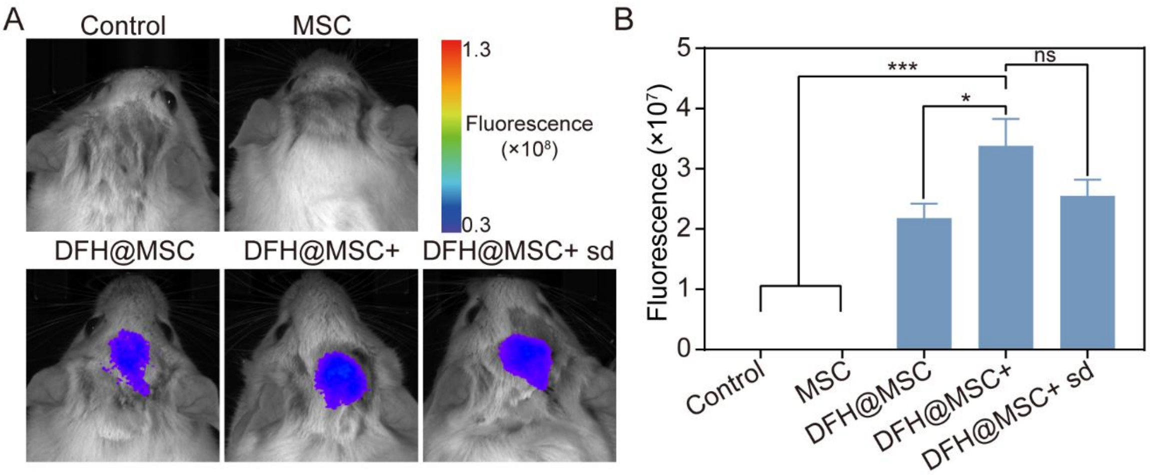


**Figure S19.** (A) *In vivo* tracking of DiR-MSC recruitment to cranial defect sites at 3 days post-implantation. (B) Quantified fluorescence intensity (FI) (n=3).

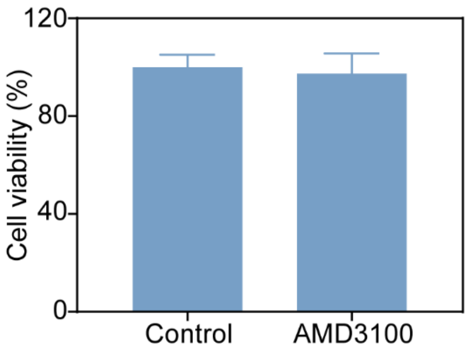


**Figure S20.** Cell viability of MSCs after 24 h treatment with AMD3100 (10 μg/mL) measured by CCK-8 assay (n=5).

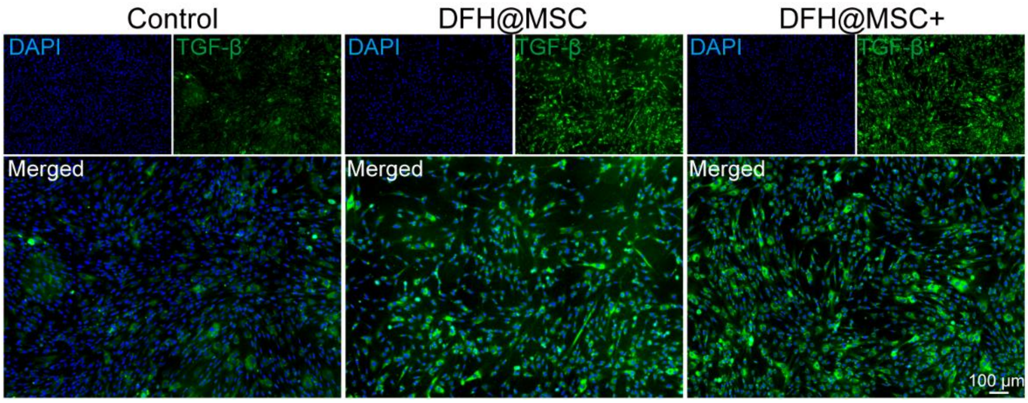


**Figure S21.** Immunofluorescence staining of TGF-β expression in DFH@MSC with or without photothermal stimulation.

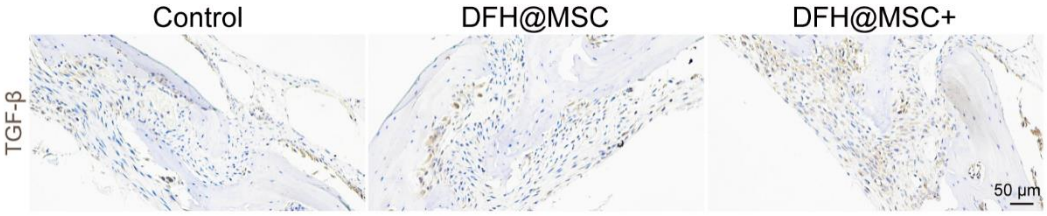


**Figure S22.** Immunohistochemical staining of TGF-β expression in cranial bone regeneration sites treated with DFH@MSC with or without photothermal stimulation.
